# Associations between perceived neighborhood environment and physical activity among breast cancer patients engaged in a physical activity program concomitant to cancer treatment: cross-sectional and longitudinal analyses in the DISCO trial (DiscoSpace)

**DOI:** 10.1186/s12966-026-01909-w

**Published:** 2026-03-26

**Authors:** Margaux Langlois, Lény Grassot, Baptiste Fournier, Olivier Trédan, Carmen Dupuis, Aurélia Maire, Annabelle Sueur, Delphine Praud, Béatrice Fervers, Olivia Pérol, Mathilde His

**Affiliations:** 1 Prevention Cancer Environment Department, Léon Bérard Cancer Center, Lyon, France; 2 Inserm U1296 Radiations: Defense, Health, Environment, Léon Bérard Cancer Center, Lyon, France; 3 Department of Medical Oncology, Léon Bérard Cancer Center, Lyon, France

**Keywords:** Breast cancer, Cancer treatment, Cross-sectional study, Longitudinal study, Perceived neighborhood environment, Physical activity

## Abstract

**Background:**

Despite the proven benefits of physical activity during breast cancer treatment, many women reduce their practice after diagnosis. A better understanding of how the neighborhood environment influences physical activity behavior could help optimize strategies for physical activity in breast cancer patients undergoing cancer treatment. This study examined associations between perceived neighborhood environment and physical activity among breast cancer patients undergoing treatment and engaged in a physical activity program.

**Methods:**

Participants were 313 breast cancer patients enrolled in the DISCO physical activity intervention trial (NCT03529383). In the present observational analysis (DiscoSpace), cross-sectional (at baseline) and longitudinal (during intervention) associations between perceived neighborhood environment and physical activity were investigated. The perceived neighborhood environment was assessed using the ALPHA questionnaire, physical activity and physical functioning were evaluated through the Recent Physical Activity Questionnaire (self-reported physical activity) and the 6-Minute Walk Test (to measure the 6-Minute Walk Distance (6MWD)). Associations were estimated through mixed linear regression models.

**Results:**

Better perceived cycling and walking infrastructures and network were associated with higher self-reported physical activity at baseline (total infrastructures: β = 0.226, 95% CI (0.063;0.388); network: β = 0.161, 95% CI (0.008;0.314)). Perceived distance to local facilities was inversely associated with 6MWD at baseline (β=-11.363, 95% CI (-20.607;-2.118)). The perception of densely populated neighborhoods (β=-0.306, 95% CI (-0.494;-0.117)) was associated with a smaller increase in self-reported physical activity during the intervention, after adjustment for trial arm. These associations varied according to women’s socioeconomic status and municipality class.

**Conclusions:**

The perceived neighborhood environment and socioeconomic characteristics of women with breast cancer should be given greater consideration for developing effective programs to promote physical activity in this population.

**Supplementary Information:**

The online version contains supplementary material available at 10.1186/s12966-026-01909-w.

## Background

Breast cancer accounts for more than one-third of new female cancer cases worldwide, representing 2.3 million incident cancers in 2022 [1]. In France, as in other high-income countries, breast cancer incidence is increasing while mortality has declined since the 1990s [2], resulting in a growing population of breast cancer patients and survivors. This population faces specific challenges during and after cancer treatments, including issues related to sequelae, comorbidities, cancer fatigue and quality of life [3], which highlights the need for comprehensive, tailored strategies to address these issues.

Physical activity during and after cancer treatment is associated with improved cancer- and non-cancer-related outcomes [4]. A positive association has been suggested with specific and overall survival in breast cancer patients [5]. Moreover, engagement in physical activity practice has been consistently associated with reduction in cancer-related fatigue and improvements in physical fitness and in the perceived quality of life in patients undergoing cancer treatment [6–8]. Several organizations have published guidelines on physical activity, emphasizing its importance in improving quality of life and reducing fatigue, especially during active cancer treatment with curative intent [4, 9, 10]. Despite this, many women reduce their practice of physical activity following breast cancer diagnosis [11, 12].

Multiple studies have investigated barriers to engaging in physical activity during and after cancer treatment [13], including the study of individual, social, and environmental factors [13, 14], with breast cancer patients being the most investigated patient group [13]. In the general population, the role of the neighborhood environment on physical activity is increasingly recognized [15–17], and this environment could even modulate the effectiveness of physical activity interventions [18]. Still, evidence on the environmental correlates of physical activity among specific populations is more limited [19]. Among cancer survivors, studies on the neighborhood environment and physical activity have yielded mixed results [20].

Among breast cancer patients specifically, two American studies have identified a negative association between the lack of facilities, or open space, and self-reported physical activity [21, 22]. In Europe, two qualitative studies also reported that limited access to recreational facilities was a significant environmental barrier to physical activity in this group [23, 24]. Further research is warranted in the European context, as urban environments in Europe differ significantly from those on other continents, particularly in terms of housing density and land use mix [25]. In addition, while objective methods, such as spatial databases (e.g., GIS), are commonly used to assess neighborhood environmental characteristics in European studies on physical activity [15], discrepancies between objectively measured (objective) and individually experienced (subjective) environmental features have been noted [26–28]. These differences highlight how objective measures may fail to capture the lived experience and the specific needs of individuals, potentially leading to inconsistent results [29, 30].

In this context, the present study aimed to examine the relationship between the perceived neighborhood environment and physical activity before treatment initiation (baseline) and change in physical activity over 6 months, among breast cancer patients engaged in a physical activity program concomitant to cancer treatment. By combining self-reported physical activity data and functional capacity measurement, this study provides a comprehensive approach to understanding how perceptions of the neighborhood environment influence physical activity in women undergoing breast cancer treatment.

## Methods

### Data collection

The current work is an ancillary study (DiscoSpace) to the phase III interventional multicenter randomized trial DISCO (NCT03529383) [31]. This controlled trial, started in 2018 and ended in 2022, was promoted by the Léon Bérard Comprehensive Cancer Center (CLB), located in Lyon, France. This trial was designed to evaluate the efficacy of a 6-months adapted physical activity program using a connected device with activity trackers and a patient therapeutic education program among women with localized breast cancer. Study rationale, methods for recruitment, enrollment, data collection, and participant characteristics of the DISCO trial have been described in details elsewhere [31]. Briefly, with a 2 × 2 factorial design (1:1:1:1 ratio), 436 women diagnosed with primary localized invasive breast carcinoma and eligible for adjuvant chemotherapy, hormonotherapy, immunotherapy and/or radiotherapy, were randomized into one of the four arms: (A) individualized exercise program carried out autonomously with a connected device, (B) therapeutic patient education sessions on physical activity, (C) both interventions, (D) control group receiving usual care. The connected device consisted of a wristband activity tracker, a smartphone application and a website, providing an individualized and adaptive program of two walking and one muscle strengthening sessions per week performed autonomously and adapted to fatigue levels through an algorithm, and evolving daily step targets. All women in the trial were informed about international recommendations on physical activity by a certified sports instructor and given a booklet. For each participant, data were collected at baseline, with follow-up assessments at 6 months and 12 months.

For the DiscoSpace study initiated in 2021, an additional self-administered questionnaire (ALPHA, Assessing Levels of PHysical Activity and Fitness at population level [25]; see Measurements paragraph for details) was sent to participants of the DISCO trial to assess their perception of the neighborhood environment at their place of residence at baseline. Depending on each patient’s status in the DISCO trial at the time of DiscoSpace initiation, the ALPHA questionnaire was administered either at inclusion in DISCO (*n* = 59 patients), during the intervention (*n* = 69 patients), or after completion of the intervention (*n* = 185 patients).

The administration of the ALPHA questionnaire for the DiscoSpace study was approved by a French ethics committee (17th November 2020). The informed consent form signed by all patients randomized in the DISCO trial mentions the possibility of re-use the study data for other research purposes.

### Study population

Of the 436 participants enrolled in the DISCO trial, all 409 patients followed at the CLB were invited to participate in the DiscoSpace study and received the French version of the ALPHA environmental questionnaire.

### Measurements

#### Perceived neighborhood environment

The standardized, self-administrated ALPHA questionnaire is a validated tool designed to measure the perceptions of the neighborhood environment (defined as “the area within approximately one kilometer or half a mile of your home or that you could walk to in 10–15 minutes” [25]) in relation to physical activity. Participants completed the questionnaire regarding their residence at baseline in the DISCO trial.

The 49 items of the long version of the ALPHA questionnaire were summed into 15 environmental scores according to the rules of the authors’ manual [32]: residential density (/315), distance to local facilities (/40), cycling infrastructure (/10), walking infrastructure (/10), total infrastructure (/20), maintenance of infrastructure (/15), safety from crime (/15), safety from traffic (/15), total safety (/30), esthetics (/15), pleasure (/20), connectivity (/15), cycling and walking network (/20), home environment (/6) and work/study environment (/10). Higher scores generally indicating a better perception of the neighborhood environment, except for residential density and distance to facilities, where higher scores reflect greater perceived residential density and longer perceived walking distances. Home and work environment scores were not analyzed as neighborhood determinants, as they did not provide relevant information about the residential environment.

#### Self-reported physical activity and functional capacity

Self-reported physical activity was assessed using the validated and standardized Recent Physical Activity Questionnaire (RPAQ) [33], in which participants described their activities over the past four weeks across different domains: commuting, occupation, leisure time, and domestic life. Only moderate-to-vigorous activities (≥ 3 Metabolic Equivalent Tasks) were considered, and scores were summed in hours per week. Data were collected at baseline and at 6-month follow-up, i.e. at the end of the intervention.

The 6-Minute Walk Distance (6MWD), which reflects an individual’s functional exercise capacity or walking ability, was objectively measured using the validated 6-Minute Walk Test (6MWT) [34]. The test records the maximum distance walked in six minutes along a flat 30-meter corridor. Assessments were conducted at baseline and 6-months by a certified professional.

#### Covariates

The trial arm corresponds to the randomization group in the DISCO trial (A/B/C/D). Clinical data, including age (years), menopausal status (pre/post), comorbidities (past or present/none), time since diagnosis (months) and since the first surgery for breast cancer (months) were collected during baseline medical assessment. Anthropometric measurements were taken by a trained professional at each visit to calculate body mass index (BMI, in kg/m^2^). Self-reported variables included educational level (≤ baccalaureate/1 to 3 years post-baccalaureate/≥ 4 years post baccalaureate), employment status after diagnosis (active/on medical leave or disabled/retired), health (score calculated from the EQ-5D-5 L questionnaire [35]), quality of life (score calculated from the EORTC QLQ-C30 questionnaire [36]), and living with a partner (yes/no). Social deprivation (yes/no) was assessed using an individual deprivation index called the “Evaluation of Deprivation and Inequalities in Health Examination Centers” (EPICES) [37], which incorporates 11 items on marital status, health insurance status, economic status, family support, and leisure activity. A cut-off score of 30, identified in a validation study of the EPICES index as the lower limit of the 4th quintile, was used to define social deprivation [38]: women with an EPICES score above this threshold were classified as deprived, while those below were considered non-deprived. The municipality class of the residence at baseline (collected through the ALPHA questionnaire) was categorized using the 3-level grid developed by the French National Institute of Statistics and Economic Studies [39]: rural municipality, large urban center, or intermediate-density municipality (*Additional File 1*). The COVID-19 pandemic trial status was determined according to the intervention period with respect to the first French lockdown, which began on March 17, 2020 (before/during or after).

### Statistical analysis

Normality of the distribution for quantitative variables was assessed graphically. Data were described using means and standard deviations (± SD) for normally distributed quantitative variables, medians and interquartile ranges (IQR) for non-normally distributed quantitative variables, and frequencies (percentages) for categorical variables. For descriptive purposes, we defined a change in BMI as a 5% change over 6 months. As suggested by previous studies, we defined a change in health as a change of 8.6 points [40] and a change in quality of life as a change of 5 points [36].

To examine the cross-sectional and longitudinal associations between perceived neighborhood environmental characteristics (predictors) and self-reported physical activity or 6MWD (dependent variables), we used linear mixed models with repeated measures of the same outcome at baseline and 6 months. The basic model was run separately for each environmental score, and included the following fixed effects: environmental score, time of visit (baseline or 6 months), and an interaction between time of visit and environmental score. A random intercept was included to account for clustering at the individual level. The main effect of the environmental score represents the association between perceived neighborhood environment and baseline physical activity (cross-sectional association), while the interaction term reflects the association between the environmental score and change in physical activity over 6 months (longitudinal association). The use of a random intercept was assessed using a likelihood ratio test to ensure the improvement of each model. Physical activity variables were modeled continuously, and square root transformation was used for self-reported physical activity to better approximate a normal distribution of residuals. For comparability of results, all environmental scores were standardized by dividing individual scores by the mean standard deviation. Standardized coefficients represent the change in the dependent variable associated with a one SD change in the predictor variable.

All models were adjusted for a priori-defined confounders selected by a Directed Acyclic Graph (*Additional File 2*). Fixed (time-invariant) confounders included age, social deprivation, educational level, employment status after diagnosis, comorbidities, living with a partner, trial arm, municipality class, perceived home environment, and the COVID-19 pandemic trial status. Time-variant confounders were measured at baseline and at 6 months, and included BMI, quality of life, and health status. Among all analyses, the one estimating the effect of residential density score on physical activity were not adjusted for municipality class because of strong correlations between these variables. Standardized regression coefficients were used to express the beta with the corresponding 95% confidence interval (CI).

Confounders, as well as other suspected modifiers, were investigated as potential effect modifiers using likelihood-ratio tests. Beforehand, time-variant variables were fixed at baseline, and quantitative variables were analyzed in subgroups using the WHO categorization for BMI [41] (< 25/≥25 kg/m²) or medians as cut-off (for age (51 years), quality of life at baseline (75/100), health status at baseline (70/100), time since diagnosis (3 months), time since the first breast cancer surgery (1 month)). For each association and each considered modifier, we first tested a two-way ANOVA interaction between the effect modifier and the explanatory variable (to model the effect of the modifier on the association between perceived neighborhood environment and physical activity at baseline), and then tested an ANOVA three-way interaction between the effect modifier, the explanatory variable, and the time-point (to model the effect of the modifier on the association over time). Statistical significance for the interaction term was set at *p* < 0.05. Results were stratified based on significant interactions.

Three sensitivity analyses were conducted to evaluate the robustness of our findings. The first adjustment accounted for the timing of the ALPHA questionnaire completion (before, during or after the DISCO trial intervention). Second, extreme values of self-reported physical activity (beyond the 99th percentile) were excluded to minimize the influence of outliers, resulting in the removal of two participants. The third sensitivity analysis stratified participants by cancer treatment subgroup (chemotherapy, immunotherapy, radiotherapy, hormonotherapy). Although cancer treatment was a potential confounder, the administration of multiple therapies during the 6-month follow-up period precluded its inclusion in the main adjustments.

Multiple Imputation by Chained Equations was applied to covariates with missing values. Under the Missing at random and Missing Completely At Random data assumptions [42], 10 imputed data sets were generated, each consisting of 10 iterations.

All the analyses were conducted using R, 4.4.0 version (notably lme4, mice and mitml packages). The type I error rate was set at 0.05.

## Results

### Population characteristics

Of the 409 patients of the DISCO trial followed at the CLB included in the DiscoSpace study, 314 completed the ALPHA environmental questionnaire (response rate 76.8%). One patient was subsequently excluded due to missing 6MWD measures at baselin*e (Flowchart available in Additional File 3).*

Baseline characteristics of the DiscoSpace study participants (*n* = 313) are shown in Table 1. The mean age at enrollment was 52.0 years old (± 10.3 years), approximately one-third (32.9%) had not completed higher education, and nearly one-half (47.9%) were on medical leave or disabled. Overall, 14.1% of the women were in a situation of social deprivation. Patients were evenly distributed among the different types of municipalities (29.7%, 33.2%, and 37.1% lived in rural areas, intermediate density municipalities and in large urban centers, respectively). Baseline characteristics of respondents and non-respondents (*n* = 95) were broadly comparable, except for social deprivation, which was more prevalent among non-respondents (21.1%). However, interpretation is limited by the high proportion of missing data among non-respondents, with 64.2% missing data on education and 63.2% on employment (*Additional File 4*).

**Table 1:** Baseline characteristics of the 313 participants of the DiscoSpace study, France, 2018–2022 (*n* = 313)

Sociodemographics	
Age (years), *mean* *±* *SD*	52.0 ± 10.3
Educational level, *n (%)*	
≤ baccalaureate	103 (32.9)
1 to 3 years post-baccalaureate	85 (27.2)
≥ 4 years post baccalaureate	92 (29.4)
Missing	33 (10.5)
Employment after diagnosis, *n (%)*	
Active	68 (21.7)
On medical leave or disabled	150 (47.9)
Retired	55 (17.6)
Missing	40 (12.8)
Social deprivation ^a^, *n (%)*	
Deprived	44 (14.1)
Non-deprived	262 (83.7)
Missing	7 (2.2)
Living with a partner, *n (%)*	
Yes	245 (78.3)
No	66 (21.1)
Missing	2 (0.6)
Health and Behaviour	
Time since diagnosis (months), *median (IQR)*	3.0 (2.5–4.0)
Missing, n (%)	2 (0.6)
Time since first surgery (months), median (IQR)	1.0 (1.0–2.0)
BMI (kg/m²), mean ± SD	25.6 ± 5.1
Missing, n (%)	1 (0.3)
BMI category, n (%)	
Normal weight	164 (52.4)
Overweight	95 (30.4)
Obesity	53 (16.9)
Missing	1 (0.3)
Quality of life (/100) ^b^, median (IQR)	75.0 (58.3–83.3)
Missing, n (%)	17 (5.4)
Health status (/100) ^c^, median (IQR)	70.0 (60.0–80.0)
Missing, n (%)	2 (0.6)
Menopausal status, n (%)	
Premenopausal or perimenopausal	166 (53.0)
Postmenopausal	145 (46.3)
Missing	2 (0.6)
Comorbidities, n (%)	
Past or present	211 (67.4)
None	102 (32.6)
Municipality class ^d^, n (%)	
Rural municipality	93 (29.7)
Intermediate-density municipality	104 (33.2)
Large urban center	116 (37.1)

*Abbreviations:* *IQR* Inter-Quartile Range, *SD* Standard Deviation

^a^ Assessed using the Evaluation of Deprivation and Inequalities in Health Examination Centers (EPICES) index, with a cut-off score of 30 to define precarity

^b^ Score calculated from the EORTC QLQ-C30 questionnaire

^c^ Score calculated from the EQ-5D-5 L questionnaire

^d^ Based on patients addresses at baseline, using the 3-level grid developed by the French National Institute of Statistics and Economic Studies (INSEE)

The characteristics of the 313 patients during the 6-month follow-up are shown in Table 2. Two thirds of patients were enrolled during or after the first national COVID-19 pandemic lockdown in France (66.1%). The vast majority of the patients received radiotherapy (90.7%) and/or hormone therapy (81.2%), approximately 57.5% received chemotherapy, and 14.1% received immunotherapy. Most participants were enrolled after the DISCO intervention (59.1%), while 22.0% were included during and 18.9% before.

**Table 2:** Intervention-related characteristics of the 313 participants of the DiscoSpace study, France, 2018–2022

Characteristics	Total (*n* = 313)
Intervention Specificities	
Trial arm, *n* (%)	
(A) Individualized, semi-supervised exercise program carried out autonomously with a connected device	79 (25.2)
(B) Therapeutic patient education sessions on physical activity	76 (24.3)
(C) Both interventions	77 (24.6)
(D) Control group receiving usual care	81 (25.9)
COVID-19 pandemic trial status, n (%)	
Before the first national lockdown	106 (33.9)
During or after the first national lockdown	207 (66.1)
Received therapies during the intervention (yes), *n (%)*	
Radiotherapy	284 (90.7)
Hormonotherapy	254 (81.2)
Chemotherapy	180 (57.5)
Immunotherapy	44 (14.1)
Observed Changes During Intervention	
Change in BMI ^a^, *n (%)*	
Weight gain	32 (10.2)
None	172 (55.0)
Weight loss	27 (8.6)
Missing	82 (26.2)
Change in health status ^b^, *n (%)*	
Improvement	114 (36.5)
None	113 (36.1)
Deterioration	58 (18.5)
Missing	28 (8.9)
Change in quality of life ^c^, *n (%)*	
Improvement	96 (30.7)
None	60 (19.2)
Deterioration	104 (33.2)
Missing	53 (16.9)

*Abbreviations: IQR* Inter-Quartile Range, *SD* Standard Deviation

^a^ Defined as a 5% increase or decrease over the 6 months

^b^ Defined as a change of ± 8.6 units over the 6 months

^c^ Defined as a change of ± 5 units over the 6 months

Data on physical activity are shown in Table 3. At the time of randomization, the median amount of self-reported physical activity was 4.1 h per week (IQR: 1.3–8.2), which increased to 8.7 h per week (IQR: 4.9–14.6) by the end of the intervention, for a median increase of + 4.6 h, and a relative increase of + 112.2%. The mean 6MWD was 575.8 m (± 77.4 m) at baseline, and 596.2 m (± 83.3 m) at 6 months, representing a median increase of + 20.4 m, and a relative increase of + 3.5%.

**Table 3:** Change in physical activity over the 6-months, DiscoSpace study, France, 2018–2022 (*n* = 313)

	Baseline, Median (IQR) / Mean ± SD	6 months, Median (IQR) / Mean ± SD	Absolute difference	Relative difference	Missing data at 6-months, *n* (%)
Self-reported physical activity (hour/week) ^a^	4.1 (1.3–8.2)	8.7 (4.9–14.6)	+ 4.6	+ 112.2%	19 (6.07)
6MWD (meters/6min) ^b^	575.8 ± 77.4	596.2 ± 83.3	+ 20.4	+ 3.5%	102 (32.6)

*Abbreviations:* *6MWD* 6-Minute Walk Distance, *IQR* Inter-Quartile Range, *SD* Standard Deviation

^a^ Self-reported physical activity was calculated from the Recent Physical Activity Questionnaire (RPAQ)

^b^ 6MWD was measured by the 6-Minute Walk Test (6MWT)

The scores obtained from the ALPHA questionnaire and their description are presented in *Additional File 5*. The infrastructure maintenance score was excluded from all the analyses due to the large number of missing values (38.3%). Other scores had minimal missing data, with residential density and distance to local facilities having the highest rates of missing responses (10.5% and 10.9%, respectively).

### Cross-sectional associations

In the overall model, an increase of one standardized unit of perceived infrastructure scores (cycling: β = 0.210, 95% CI (0.048;0.372); walking: β = 0.170, 95% CI (0.016;0.324); total infrastructure: β = 0.226, 95% CI (0.063;0.388), per 1SD) and walking and cycling network score (β = 0.161, 95% CI (0.008;0.314), per 1SD) predicted higher levels of self-reported physical activity at baseline (Table 4). Moreover, an increase of one standardized unit of the perceived distance to facilities was associated with lower 6MWD at baseline (-11 m; β=-11.363, 95% CI (-20.607;-2.118), per 1SD). No association was observed for all other environmental scores and 6MWD at baseline.

**Table 4:** Associations between perceived neighborhood environment and physical activity, DiscoSpace study, France, 2018–2022 (*n* = 313)

Perceived neighborhood environment ^a^	Physical Activity Outcome					
	Self-reported physical activity ^b^			6MWD ^c^		
	β ^d^	95% CI	*p*-value	β ^d^	95% CI	*p*-value
Residential density						
Cross-sectional ^e^	0.047	(-0.105 ; 0.199)	0.542	0.189	(-8.377 ; 8.755)	0.965
Longitudinal ^f^	**-0.306**	**(-0.494 ; -0.117)**	**0.002**	2.307	(-6.163 ; 10.776)	0.593
Distance to local facilities						
Cross-sectional ^e^	-0.071	(-0.242 ; 0.100)	0.413	**-11.363**	**(-20.607 ; -2.118)**	**0.016**
Longitudinal ^f^	0.181	(-0.014 ; 0.377)	0.069	0.160	(-8.379 ; 8.700)	0.971
Cycling infrastructures						
Cross-sectional ^e^	**0.210**	**(0.048 ; 0.372)**	**0.011**	4.509	(-4.081 ; 13.098)	0.303
Longitudinal ^f^	-0.151	(-0.340 ; 0.038)	0.117	0.058	(-7.725 ; 7.841)	0.988
Walking infrastructures						
Cross-sectional ^e^	**0.170**	**(0.016 ; 0.324)**	**0.031**	1.685	(-6.376 ; 9.747)	0.681
Longitudinal ^f^	-0.159	(-0.348 ; 0.030)	0.099	0.815	(-7.023 ; 8.653)	0.838
Total infrastructures						
Cross-sectional ^e^	**0.226**	**(0.063 ; 0.388)**	**0.007**	3.837	(-4.806 ; 12.479)	0.384
Longitudinal ^f^	-0.175	(-0.363 ; 0.013)	0.068	0.988	(-7.338 ; 8.208)	0.912
Safety from crime						
Cross-sectional ^e^	-0.076	(-0.235 ; 0.083)	0.347	-0.979	(-9.288 ; 7.330)	0.817
Longitudinal ^f^	0.113	(-0.075 ; 0.301)	0.240	-3.964	(-11.802 ; 3.874)	0.321
Safety from traffic						
Cross-sectional ^e^	-0.009	(-0.155 ; 0.138)	0.906	5.344	(-2.126 ; 12.815)	0.160
Longitudinal ^f^	0.138	(-0.049 ; 0.326)	0.148	-6.353	(-14.044 ; 1.338)	0.105
Total safety						
Cross-sectional ^e^	-0.040	(-0.193 ; 0.114)	0.610	3.224	(-4.727 ; 11.175)	0.426
Longitudinal ^f^	0.147	(-0.041 ; 0.335)	0.125	-6.114	(-13.901 ; 1.673)	0.124
Esthetics						
Cross-sectional ^e^	0.017	(-0.139 ; 0.173)	0.828	4.879	(-4.252 ; 12.010)	0.349
Longitudinal ^f^	0.152	(-0.037 ; 0.340)	0.114	-7.150	(-15.223 ; 0.923)	0.082
Pleasure						
Cross-sectional ^e^	0.032	(-0.125 ; 0.188)	0.692	6.207	(-1.959 ; 14.373)	0.136
Longitudinal ^f^	0.174	(-0.015 ; 0.364)	0.071	-6.862	(-15.052 ; 1.328)	0.100
Connectivity						
Cross-sectional ^e^	0.120	(-0.030 ; 0.269)	0.118	4.457	(-3.252 ; 12.166)	0.257
Longitudinal ^f^	-0.049	(-0.241 ; 0.144)	0.620	-3.482	(-11.676 ; 4.713)	0.404
Walking and cycling network						
Cross-sectional ^e^	**0.161**	**(0.008 ; 0.314)**	**0.039**	2.630	(-5.290 ; 10.551)	0.514
Longitudinal ^f^	-0.140	(-0.331 ; 0.050)	0.148	-3.191	(-11.301 ; 4.919)	0.440

Values in bold are statistically significant (*P* < 0.05)

^a^ Environmental scores were calculated from the ALPHA questionnaire (for Assessing Levels of PHysical Activity and Fitness at population level)

^b^ Self-reported physical activity was calculated from the Recent Physical Activity Questionnaire (RPAQ). The average difference in the outcome self-reported physical activity is expressed by the square root

^c^ 6MWD was measured by the 6-Minute Walk Test (6MWT). The average difference in the outcome 6MWD is expressed without transformation

^d^ The β indicate the overall longitudinal difference in the outcome score using linear mixed models per 1 SD of perceived built environment score after a standardized Z-score transformation. Analyses were adjusted on: age, social deprivation, educational level, employment status after diagnosis, comorbidities, living with a partner, trial arm, municipality class (except for Residential density score analyses), perceived home environment, COVID-19 pandemic trial status, longitudinal BMI, longitudinal quality of life, and longitudinal health status

^e^ The cross-sectional association of perceived neighborhood environment and physical activity is estimated by the environmental perception score term

^f^ The longitudinal association of perceived neighborhood environment and physical activity is estimated by the interaction term between the intervention visit and the environmental perception score

### Longitudinal associations

Overall, an increase in physical activity from baseline to 6-month follow-up was observed after adjustments for trial arm (mean effect of time on self-reported physical activity: β > 0.900, mean effect of time on 6MWD: β > 23.228; *p* < 0.001) (*Additional File 6*). There was a negative association between perceived residential density and the change in self-reported physical activity (β=-0.306, 95% CI (-0.494;-0.117), per 1SD) (Table 4). In contrast, no significant association was found between the perceived neighborhood environment and the change in 6MWD from baseline to 6 months.

### Stratification by social deprivation

After stratification by social deprivation, positive associations between baseline self-reported physical activity and perceived infrastructures remained only among non-deprived patients (with β = 0.209, 95% CI (0.038;0.379) for cycling; β = 0.204, 95% CI (0.037;0.371) for walking; β = 0.240, 95% CI (0.068;0.413) for total infrastructures, per 1SD); although interactions were not statistically significant for walking and total infrastructure scores (Table 5).

Additionally, inverse associations between perceived residential density and self-reported physical activity change remained only among non-deprived patients (β=-0.400, 95% CI (-0.602;-0.195), per 1SD; p-value for interaction = 0.031). Moreover, among non-deprived women, positive associations were observed (per 1SD increase) between change in self-reported physical activity and distance to local facilities (β = 0.298, 95% CI (0.085;0.511)), and perceived infrastructures (β=-0.242, 95% CI (-0.442;-0.041) for cycling; β=-0.283, 95% CI (-0.487;-0.079) for walking; β=-0.292, 95% CI (-0.492;-0.092) for total infrastructures) (p-values for interaction ≤ 0.05). A positive association between self-reported physical activity change and perceived safety form traffic appeared among deprived women (β = 0.632, 95% CI (0.196;1.068), per 1SD; p-value for interaction = 0.016), but not among non-deprived women (β = 0.034, 95% CI (-0.175;0.243), per 1SD) (Table 5).

An inverse association between the perceived safety from crime and 6MWD at baseline was observed among deprived women only (β=-23.860, 95% CI (-41.601;-6.118), per 1SD). Significant positive associations between baseline 6MWD and connectivity (β = 24.060, 95% CI (3.533;44.587), per 1SD), as well as cycling and walking network (β = 19.466, 95% CI (1.004;37.928), per 1SD) were observed among patients experiencing social deprivation but not among others (Table 5).

**Table 5:** Associations between perceived neighborhood environment and physical activity, stratified by social deprivation, DiscoSpace study (*n* = 306)

Perceived neighborhood environment score ^a^	Non-deprived (*n* = 262)			Deprived (*n* = 44)			*p*-int
	β ^c^	95% CI	*p*-value	β ^c^	95% CI	*p*-value	
**Self-reported physical activity** ^**b**^							
Residential density							
Cross-sectional ^e^	0.083	(-0.079 ; 0.246)	0.314	-0.154	(-0.555 ; 0.246)	0.449	0.724
Longitudinal ^f^	**-0.400**	**(-0.602 ; -0.195)**	**< 0.001**	0.206	(-0.316 ; 0.729)	0.438	**0.031**
Distance to local facilities							
Cross-sectional ^e^	-0.100	(-0.286 ; 0.087)	0.293	0.087	(-0.276 ; 0.451)	0.637	0.444
Longitudinal ^f^	**0.298**	**(0.085 ; 0.511)**	**0.006**	-0.187	(-0.582 ; 0.208)	0.352	**0.011**
Cycling infrastructures							
Cross-sectional ^e^	**0.209**	**(0.038 ; 0.379)**	**0.017**	0.219	(-0.189 ; 0.626)	0.292	**0.044**
Longitudinal ^f^	**-0.242**	**(-0.442 ; -0.041)**	**0.018**	0.446	(-0.086 ; 0.977)	0.100	**0.019**
Walking infrastructures							
Cross-sectional ^e^	**0.204**	**(0.037 ; 0.371)**	**0.017**	0.005	(-0.373 ; 0.383)	0.981	0.319
Longitudinal ^f^	**-0.283**	**(-0.487 ; -0.079)**	**0.007**	0.457	(-0.029 ; 0.943)	0.065	**0.006**
Total infrastructures							
Cross-sectional ^e^	**0.240**	**(0.068 ; 0.413)**	**0.006**	0.135	(-0.263 ; 0.533)	0.506	0.085
Longitudinal ^f^	**-0.292**	**(-0.492 ; -0.092)**	**0.004**	0.502	(-0.009 ; 1.013)	0.054	**0.005**
Safety from traffic							
Cross-sectional ^e^	0.041	(-0.120 ; 0.202)	0.619	0.258	(-0.600 ; 0.083)	0.138	0.969
Longitudinal ^f^	0.034	(-0.175 ; 0.243)	0.750	**0.632**	**(0.196 ; 1.068)**	**0.005**	**0.016**
**6MWD** ^**c**^							
Safety from crime							
Cross-sectional ^e^	4.210	(-4.758 ; 13.179)	0.357	**-23.860**	**(-41.601 ; -6.118)**	**0.008**	**0.008**
Longitudinal ^f^	-6.774	(-15.568 ; 2.020)	0.131	7.476	(-11.620 ; 26.572)	0.442	0.171
Total safety							
Cross-sectional ^e^	7.304	(-1.279 ; 15.887)	0.095	-16.271	(-34.291 ; 1.748)	0.077	**0.033**
Longitudinal ^f^	-8.648	(-17.382 ; 0.085)	0.052	4.418	(-16.138 ; 24.974)	0.673	0.230
Connectivity							
Cross-sectional ^e^	1.439	(-6.802 ; 9.681)	0.732	**24.060**	**(3.533 ; 44.587)**	**0.022**	**0.048**
Longitudinal ^f^	-2.275	(-11.219 ; 6.669)	0.618	-11.285	(-32.979 ; 10.409)	0.307	0.447
Walking and cycling network							
Cross-sectional ^e^	-0.733	(-9.325 ; 7.860)	0.867	**19.466**	**(1.004 ; 37.928)**	**0.039**	**0.049**
Longitudinal ^f^	-2.099	(-11.117 ; 6.919)	0.648	-8.964	(-28.567 ; 10.640)	0.369	0.529

Values in bold are statistically significant (*P* < 0.05) ; Social deprivation score was assessed using an individual deprivation index called the “Evaluation of Deprivation and Inequalities in Health Examination Centers” (EPICES) and a cut-off score of 30 was used to define social deprivation (yes/no)

^a^ Environmental scores were calculated from the ALPHA questionnaire (for Assessing Levels of PHysical Activity and Fitness at population level)

^b^ Self-reported physical activity was calculated from the Recent Physical Activity Questionnaire (RPAQ). The average difference in the outcome self-reported physical activity is expressed by the square root

^c^ 6MWD was measured by the 6-Minute Walk Test (6MWT). The average difference in the outcome 6MWD is expressed without transformation

^d^ The β indicate the overall longitudinal difference in the outcome score using linear mixed models per 1 SD of perceived built environment score after a standardized Z-score transformation. Analyses were adjusted on: age, social deprivation, educational level, employment status after diagnosis, comorbidities, living with a partner, trial arm, municipality class (except for Residential density score analyses), perceived home environment, COVID-19 pandemic trial status, longitudinal BMI, longitudinal quality of life, and longitudinal health statu

^e^ The cross-sectional association of perceived neighborhood environment and physical activity is estimated by the environmental perception score term ; ^f^ The longitudinal association of perceived neighborhood environment and physical activity is estimated by the interaction term between the intervention visit and the environmental perception score

### Stratification by municipality class

After stratification by municipality class, associations between self-reported physical activity at baseline and perceived infrastructure remained only among rural municipalities (β = 0.512, 95% CI (0.202;0.822) for cycling; β = 0.448 95% CI (0.155;0.740) for total infrastructures, per 1SD) (Table 6).

**Table 6:** Associations between perceived neighborhood environment and physical activity, stratified by municipality class, DiscoSpace study (*n* = 313)

Perceived neighborhood environment ^a^	Self-reported physical activity ^b^									
	Rural municipality (*n* = 93)			Intermediate-density municipality (*n* = 104)			Large urban center (*n* = 116)			*p*-int
	β ^c^	95% CI	*p*-value	β ^c^	95% CI	*p*-value	β ^c^	95% CI	*p*-value	
Distance to local facilities										
Cross-sectional ^d^	-0.219	(-0.500 ; 0.062)	0.126	-0.002	(-0.303 ; 0.299)	0.990	0.271	(-0.121 ; 0.664)	0.175	0.122
Longitudinal ^e^	0.175	(-0.189 ; 0.539)	0.346	**0.427**	**(0.037 ; 0.818)**	**0.032**	**-0.565**	**(-1.066 ; -0.065)**	**0.027**	**0.008**
Cycling infrastructures										
Cross-sectional ^d^	**0.512**	**(0.202 ; 0.822)**	**0.001**	0.040	(-0.236 ; 0.316)	0.775	-0.028	(-0.345 ; 0.289)	0.862	0.302
Longitudinal ^e^	**-0.568**	**(-0.993 ; -0.143)**	**0.009**	-0.057	(-0.410 ; 0.295)	0.750	**0.416**	**(0.008 ; 0.825)**	**0.046**	**0.004**
Total infrastructures										
Cross-sectional ^d^	**0.448**	**(0.155 ; 0.740)**	**0.003**	0.073	(-0.199 ; 0.346)	0.598	0.013	(-0.331 ; 0.357)	0.940	0.407
Longitudinal ^e^	**-0.525**	**(-0.912 ; -0.139)**	**0.008**	-0.090	(-0.438 ; 0.258)	0.611	0.440	(-0.001 ; 0.882)	0.050	**0.005**
Connectivity										
Cross-sectional ^d^	0.127	(-0.123 ; 0.376)	0.319	0.208	(-0.038 ; 0.454)	0.098	-0.144	(-0.450 ; 0.162)	0.356	0.707
Longitudinal ^e^	0.067	(-0.265 ; 0.398)	0.693	**-0.357**	**(-0.682 ; -0.032)**	**0.031**	**0.535**	**(0.126 ; 0.943)**	**0.010**	**0.003**
Walking and cycling network										
Cross-sectional ^d^	0.221	(-0.049 ; 0.490)	0.108	0.185	(-0.064 ; 0.434)	0.146	-0.056	(-0.360 ; 0.248)	0.718	0.234
Longitudinal ^e^	-0.037	(-0.392 ; 0.318)	0.839	**-0.455**	**(-0780 ; -0.130)**	**0.006**	**0.493**	**(0.083 ; 0.903)**	**0.018**	**0.002**

Values in bold are statistically significant (*P* < 0.05) ; The municipality class of the residence at baseline was categorized using the 3-level grid developed by the French National Institute of Statistics and Economic Studies (INSEE)

^a^ Environmental scores were calculated from the ALPHA questionnaire (for Assessing Levels of PHysical Activity and Fitness at population level)

^b^ Self-reported physical activity was calculated from the Recent Physical Activity Questionnaire (RPAQ). The average difference in the outcome self-reported physical activity is expressed by the square root

^c^ The β indicate the overall longitudinal difference in the outcome score using linear mixed models per 1 SD of perceived built environment score after a standardized Z-score transformation. Analyses were adjusted on: age, social deprivation, educational level, employment status after diagnosis, comorbidities, living with a partner, trial arm, municipality class (except for Residential density score analyses), perceived home environment, COVID-19 pandemic trial status, longitudinal BMI, longitudinal quality of life, and longitudinal health status

^d^ The cross-sectional association of perceived neighborhood environment and physical activity is estimated by the environmental perception score term

^e^ The longitudinal association of perceived neighborhood environment and physical activity is estimated by the interaction term between the intervention visit and the environmental perception score

After stratification, significant associations were observed: perceived shorter distances to local facilities (β=-0.565, 95% CI (-1.066;-0.065), per 1SD), better connectivity (β = 0.535, 95% CI (0.126;0.943), per 1SD), and a better cycling and walking network (β = 0.493, 95% CI (0.083;0.903), per 1SD), were associated with a greater increase in self-reported physical activity over 6-months in large urban centers, which contrasts with the direction of associations observed in intermediate-density municipalities (respectively: β = 0.427, 95% CI (0.037;0.818); β=-0.357, 95% CI (-0.682;-0.032); β = -0.455, 95% CI (-0780;-0.130), per 1SD). Moreover, perceived cycling infrastructure was associated with a smaller increase at 6-months in self-reported physical activity in rural municipalities (β=-0.568, 95% CI (-0.993;-0.143), per 1SD), but with a greater increase in large urban centers (β = 0.416, 95% CI (0.008;0.825), per 1SD) (Table 6).

### Additional modifiers

Regarding other modifiers, for self-reported physical activity, significant interactions were observed at baseline for employment status after diagnosis, quality of life, and trial arm; and in longitudinal analyses for BMI and health status. For 6MWD, significant interactions were observed in cross-sectional analyses for age, employment status after diagnosis, living with a partner, and trial arm; and in longitudinal analyses for time since first breast cancer surgery, quality of life, living with a partner, and trial arm (*Additional File 7*). However, most of the time, confidence intervals overlap between subgroups and/or subgroup associations were not significant.

### Sensitivity analyses

The sensitivity analysis adjusted for the ALPHA questionnaire completion period did not show any significant differences in the associations studied (*Additional File 8)*. After removing extreme values of self-reported physical activity, direction and strength of observed associations remained unchanged (*Additional File 9*). Generally, the associations observed in the main analysis were consistent after stratification by treatment subgroups, although some of them disappeared in certain subgroups (*Additional File 10*).

## Discussion

To the best of our knowledge, this study is the first to perform a quantitative analysis of the effect of perceived neighborhood environment on physical activity in women with breast cancer. The perception of developed walking and cycling infrastructures and network was associated with higher self-reported physical activity at baseline. Furthermore, patients who reported being distant from local facilities exhibited lower initial 6MWD. While there was a general increase in self-reported physical activity over the six-month study period after adjustments for trial arm, this increase was more modest in neighborhoods perceived to be densely populated. Moreover, among women experiencing social deprivation, a heightened sense of traffic safety was associated with a more substantial increase in self-reported physical activity over the 6-month study period. Among non-deprived women, perceived better infrastructures and higher residential density were associated with smaller increases in self-reported physical activity over the study period. The associations between the perception of the neighborhood environment (distance to local facilities, walking and biking infrastructures and network) and changes in self-reported physical activity further varied according to the size of the municipality of residence.

### Residential density

Greater perceived residential density was associated with smaller improvements in self-reported physical activity during the intervention, after adjusting for trial arms, despite no association being observed with baseline self-reported activity levels. The literature on this topic is inconsistent: two cross-sectional studies, one among Spanish patients with chronic obstructive pulmonary disease [43] and the other among Chinese adults from the general population [44], reported that higher residential density was associated with increased sedentary behavior. Conversely, an Australian study found that urban densification was linked to greater engagement in active behaviors [45]. In densely populated areas, increased air pollution—as shown in American and Chinese studies [46–48]—may discourage outdoor activities, potentially limiting improvements in overall activity levels. Additionally, higher traffic volumes [49] and noise annoyance [50], as reported in European longitudinal studies, might further reduce participation in physical activity.

### Distance to facilities

In the present study, patients who perceived a greater distance to reach neighborhood facilities (e.g., shops, restaurants, public transport, leisure facilities, etc.) had a lower 6MWD at baseline. This supports the hypothesis that perceiving facilities as distant may encourage motorized transport over active travel, thereby reducing walking capacity. This finding is consistent with a systematic review [51] showing that convenient distance to multiple destinations is associated with favorable cardiorespiratory fitness. Our findings may also suggest that patients with lower functional capacity may perceive facilities as being farther away. For example, an American study reported that elderly and individuals with chronic pain or reduced mobility tend to perceive distances as farther compared to pain-free or higher-capacity individuals [52].

Our results showed that increased perceived distance to access facilities was associated with greater increases in physical activity during intervention among patients in intermediate-sized municipalities, and the opposite association was observed among patients in large urban centers. In smaller communities, the perception of distance may be seen as a challenge to overcome, encouraging patients to be more active to access available resources. In contrast, in urban centers, where facilities are closer and more accessible, patients may not feel the same need to engage in physical activity. Given the established tendency of urban populations to utilize the proximity of destinations for their physical activity [53], an alternative hypothesis suggests that they have already reached a state of maximum potential in this regard. In contrast, patients residing in smaller municipalities may be compelled to explore novel strategies for enhancing exercise, such as the utilization of active transportation to reach their destinations.

### Walkability and bikeability

Consistent with previous studies conducted on adult populations with or without a cancer history [17, 20, 54, 55], our findings showed that overall walkability and bikeability–as proxied in our research by perceptions of infrastructure (such as bike lanes, sidewalks and pedestrian zones for shopping) and street connectivity (including intersections and shortcuts by foot or bike)–were associated with higher levels of self-reported physical activity at baseline. However, walkability and bikeability, often used to summarize the built environment, lack consistent definitions [56, 57] and fail to capture specific environmental characteristics. Indeed, walking and cycling networks, walking and cycling infrastructures, residential density, or safety may be differently perceived and associated with physical activity pattern, as shown in our analysis.

#### Walking and cycling network

For walking and cycling network, differences were observed between deprived and non-deprived patients. Deprived patients who perceived streets to be more interconnected—referring to perceived connectivity as well as cycling and walking networks—exhibited higher baseline 6MWD. This observation suggests that individuals, even those from deprived backgrounds, may benefit from well-designed environments, potentially mitigating socioeconomic inequalities in physical functioning. However, when examining self-reported physical activity, no significant associations were observed between environmental characteristics and baseline physical activity among deprived women. Previous Canadian studies have reported mixed results, with some studies showing positive associations [58] and others negative associations [59] between street connectivity and physical activity in adults of low socioeconomic status. These discrepancies may stem from variations in the measurement of socioeconomic status (often determined by household income and/or educational level) or the specific cultural and geographic context.

#### Walking and cycling infrastructures

In the present study, after adjustment for intervention arm, the availability of walking and cycling infrastructure was associated with smaller improvements in self-reported physical activity over the 6-month study period, but only among non-deprived women. This contrasts with previous studies showing a positive association between neighborhood amenities [60, 61], access to public transport [62], and physical activity among women wishing to be more active. However, none of the aforementioned studies focused on European settings nor breast cancer patients. A Canadian study on prostate cancer patients [63] found that the neighborhood environment was not associated with physical activity patterns in a behavior change intervention. This study did not distinguish between deprived patients and others, which might explain the differences with our results.

Furthermore, the present study suggests a negative association between the perception of cycling infrastructure and improvements in physical activity in rural municipalities, and a positive association in urban areas, where infrastructures are more likely to be integrated into the daily environment. In rural areas, as highlighted by other research [64], the perception of infrastructure is often related to factors such as accessibility or safety, which may limit its benefit on physical activity. These findings underscore the importance of considering the surrounding environmental context which can influence the effectiveness of existing infrastructures in promoting physical activity. Indeed, recent studies [27] demonstrated how neighborhood perceptions can shed light on specific concerns of different groups regarding social and neighborhood contexts, and how enhancing the neighborhood environment alone may not be enough to increase physical activity.

#### Safety

In our population of breast cancer patients, deprived women who perceived their surroundings as safe regarding crime exhibited lower baseline 6MWD, even when accounting for health statuses and BMI. This result contradicts studies conducted in general adult populations [17, 65], which have established a positive association between walking and personal safety, especially when it was based on subjective measures. While the difference in results could be partly explained by the relatively small number of deprived women in our sample, future studies focusing specifically on a sample of deprived breast cancer patients may provide deeper insights into this association. Indeed, the persistence of other socioeconomic barriers, such as limited financial resources, restricted access to healthcare, or greater family and work-related responsibilities, has the potential to impact the use of the environment for physical activity [66].

Our work also revealed that, although no association was observed in the overall population, among deprived women, a higher perception of traffic safety was linked to greater improvements in self-reported physical activity over time. Previous studies [18] have shown mixed evidence regarding the impact of neighborhood environment characteristics on physical activity change in adults, depending on the population and type of physical activity program involved. This variability underscores the necessity to consider perceived neighborhood environment in futures studies by accounting for the potential influence of socioeconomic status on these relationships.

### Strengths and limitations

The DISCO trial provided accurate and longitudinal measurements of physical activity and clinical factors. Another strength is the use of a validated tool to assess environmental perceptions in a European context [67].

However, the present study is not without limitations. Although social deprivation, educational level, and municipality class varied within the sample, the generalizability of our findings is limited by the recruitment from a single cancer center in the Auvergne-Rhône-Alpes French region, with potential underrepresentation of deprived patients [68] and selection bias due to voluntary participation in a physical activity intervention [69]. However, the absence of differences in associations between trial arms suggests a limited risk of bias related to the use of DISCO study data. Moreover, most of the patients completed the ALPHA environmental questionnaire months after the 6-month physical activity assessment, which limits causal inference. Although adjustment for the time period of ALPHA questionnaire completion did not modify our findings and indications of heterogeneity between intervention arms were limited, residual bias cannot be completely ruled out. Finally, self-reported physical activity (RPAQ) may also be overestimated due to social desirability bias. However, parallel analyses on objective 6-Minute Walk Distance (6MWD) strengthened the findings and showed that perceived environmental characteristics were differentially associated with self-reported activity and 6MWD, highlighting the value of analyzing both dimensions [70].

## Conclusions

The presence of adequate infrastructures and the development of a comprehensive cycling and walking network were positively associated with baseline self-reported physical activity in this population of breast cancer patients engaged in physical activity. Furthermore, the associations between the perceived neighborhood environment and improvements in both self-reported physical activity and functional capacity (6MWD) varied according to individual socioeconomic status (social deprivation) and municipality class, after adjusting for the physical activity program followed during the DISCO trial. These findings underscore the necessity to further consider both individual socioeconomic characteristics and perceived environmental characteristics when designing tailored physical activity interventions for breast cancer patients. In addition, individual-level strategies need to be complemented by broader structural and environmental actions aimed at improving neighbourhood conditions, enhancing perceptions of patients’ local environment, and addressing socioeconomic inequalities, in order to create supportive contexts for sustained physical activity.

## Supplementary Information

Supplementary Material 1.

Supplementary Material 2.

Supplementary Material 3.

Supplementary Material 4.

Supplementary Material 5.

Supplementary Material 6.

Supplementary Material 7.

Supplementary Material 8.

Supplementary Material 9.

Supplementary Material 10.

Supplementary Material 11.

## Data Availability

The datasets supporting the conclusions of this article are available from the corresponding author on reasonable request. For general data sharing inquiries, contact Béatrice Fervers ( [beatrice.fervers@lyon.unicancer.fr](mailto: beatrice.fervers@lyon.unicancer.fr) ).

## Abbreviations

6MWD: 6-Minute Walk Distance
6MWT: 6-Minute Walk Test
ALPHA: Assessing Levels of PHysical Activity and Fitness at population level
BMI: Body Mass Index
CLB: Léon Bérard Comprehensive Cancer Center
CI: Confidence Interval
DAG: Directed Acyclic Graph
DISCO: DISpositif COnnecté
EPICES: Evaluation of Deprivation and Inequalities in Health Examination Centers
IQR: Inter-Quartile Range
RPAQ: Recent Physical Activity Questionnaire
SD: Standard Deviation
WHO: World Health Organization
