# Supplementary material for: Associations between perceived neighborhood environment and physical activity among breast cancer patients engaged in a physical activity program concomitant to cancer treatment: cross-sectional and longitudinal analyses in the DISCO trial (DiscoSpace)
Source: Int J Behav Nutr Phys Act. 2026 Mar 26;23:48. doi: 10.1186/s12966-026-01909-w (PMC13154525; doi:10.1186/s12966-026-01909-w)
Supplement: Supplementary file 1 — Supplementary Material 1. [file 12966_2026_1909_MOESM1_ESM.docx]

**Additional File 1** – Creation and definition of the municipality class variable, DISCO-SPACE study, France, 2018-2022

**Translation from The National Institute of Statistics and Economic Studies (Insee) website (https://www.insee.fr/fr/information/6439600)**

The (French) municipal density grid classifies municipalities based on their population size and the distribution of residents across their territory. The more numerous and concentrated the population is, the denser the municipality is considered.

Belonging to a specific level of the grid is not solely determined by the average population density calculated over the entire municipality (including uninhabited areas such as forests, mountains, and fields). Instead, it also considers the presence of zones within the municipality that concentrate a large number of residents in a small area.

More specifically, the definition adopted by the European Union relies on dividing the territory into 1-kilometer-square tiles and aggregating tiles with similar density to form “patches” or “clusters”^1^. Each municipality is then assigned to a category based on the proportion of its population located within the different types of clusters.

Population data are sourced from demographic files on housing and individuals (Fidéli 2018^2^).

At its first level, with three categories, the municipal density grid distinguishes three types of municipalities:

- Densely populated municipalities;
- Intermediate-density municipalities;
- Rural municipalities.

Densely populated municipalities and intermediate-density municipalities together form the urban area and are referred to as "urban municipalities."

**References :**

1. European Commission. Statistical Office of the European Union. *Applying the Degree of Urbanisation: A Methodological Manual to Define Cities, Towns and Rural Areas for International Comparisons : 2021 Edition.* (Publications Office, LU, 2021).

2. Insee & Ministère Des Finances (DGFiP). Fichiers Démographiques sur les Logements et les Individus - 2018. Centre d’Accès Sécurisé aux Données (CASD) https://doi.org/10.34724/CASD.295.3257.V1 (2020).
