# Supplementary material for: Associations between perceived neighborhood environment and physical activity among breast cancer patients engaged in a physical activity program concomitant to cancer treatment: cross-sectional and longitudinal analyses in the DISCO trial (DiscoSpace)
Source: Int J Behav Nutr Phys Act. 2026 Mar 26;23:48. doi: 10.1186/s12966-026-01909-w (PMC13154525; doi:10.1186/s12966-026-01909-w)
Supplement: Supplementary file 2 — Supplementary Material 2. [file 12966_2026_1909_MOESM2_ESM.docx]

**Additional File 2** – Directed Acyclic Graph, DISCO-SPACE study, France, 2018-2022.


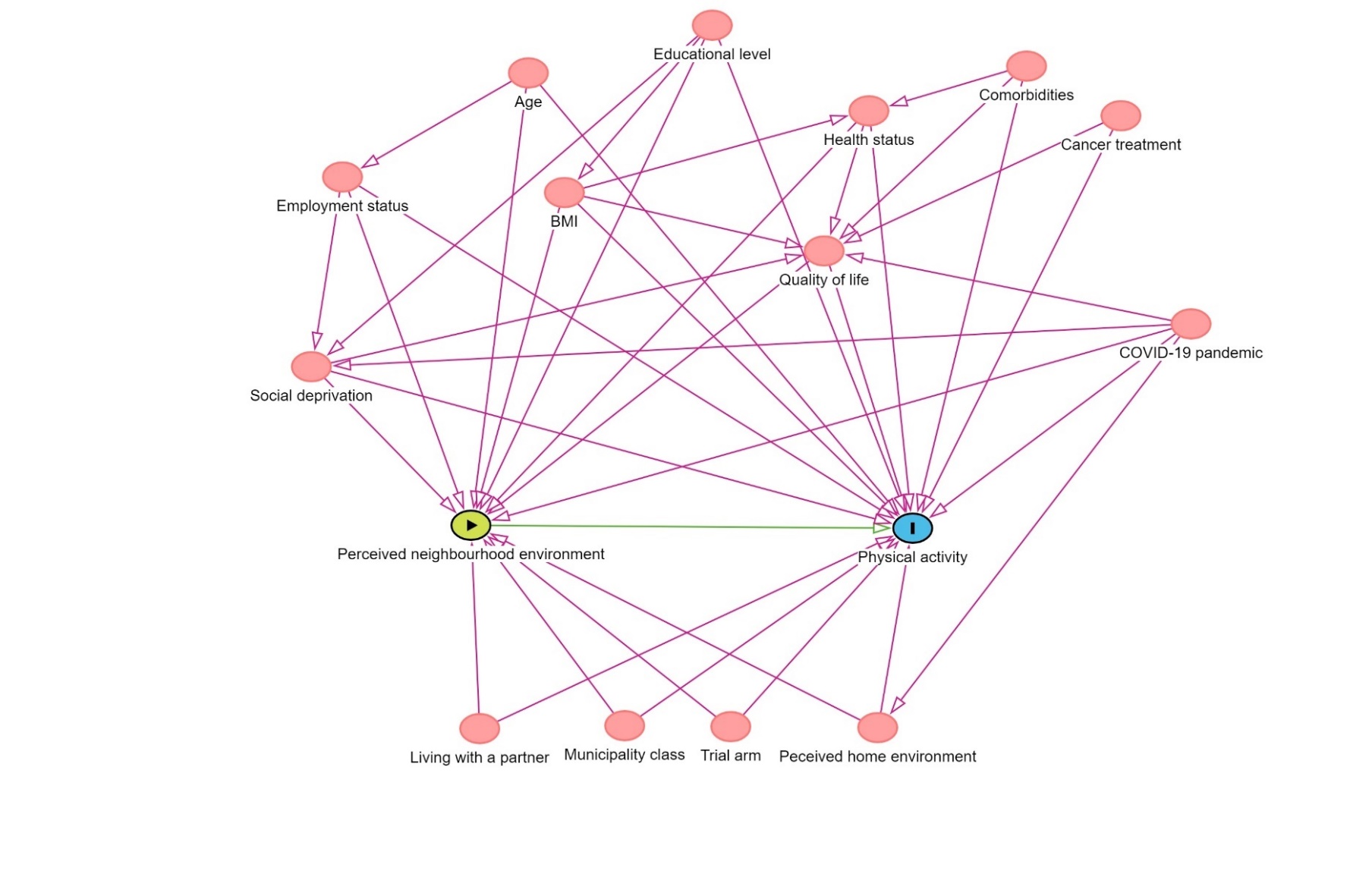


The Diagram Acyclic Graph (DAG) identifies the minimal sufficient adjustment set necessary for estimating the effect of the perceived neighborhood environment on physical activity among breast cancer patients undergoing treatment.

This DAG was used in each analysis, except for those estimating the effect of residential density score (S1) on physical activity which were not adjusted for municipality class (because of strong correlations between these variables).
