# Supplementary figures and images for: Associations between perceived neighborhood environment and physical activity among breast cancer patients engaged in a physical activity program concomitant to cancer treatment: cross-sectional and longitudinal analyses in the DISCO trial (DiscoSpace)
Source: Int J Behav Nutr Phys Act. 2026 Mar 26;23:48. doi: 10.1186/s12966-026-01909-w (PMC13154525; doi:10.1186/s12966-026-01909-w)

**Additional File 3** – Flowchart for participant inclusion, DISCO-SPACE study, France, 2018-2022.

**
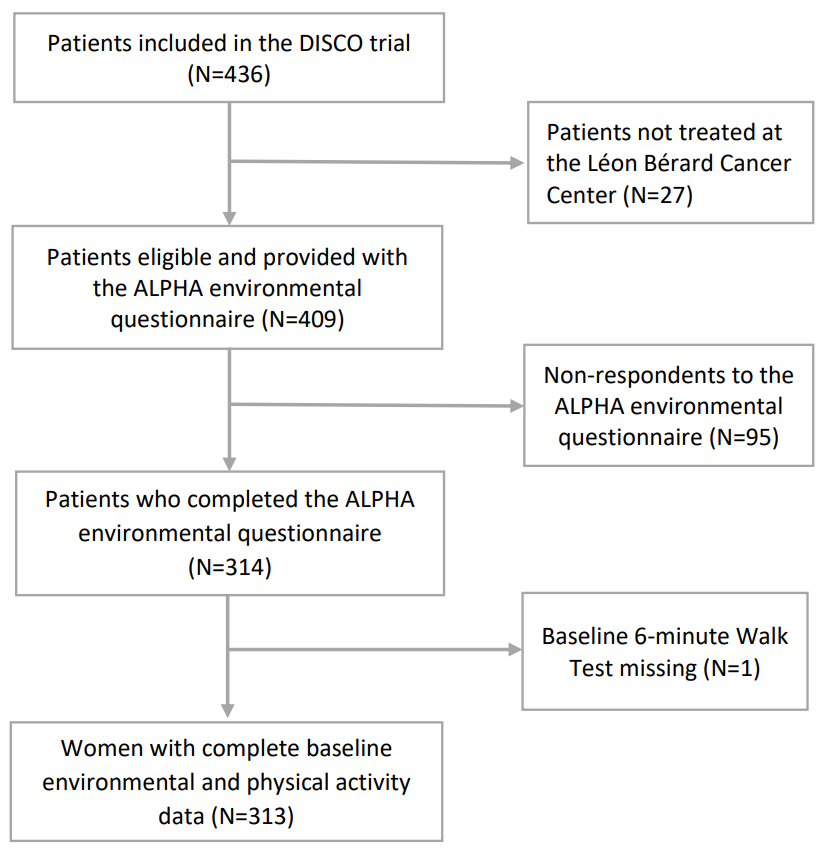
**

Supplement: Supplementary file 3 — Supplementary Material 3. [file 12966_2026_1909_MOESM3_ESM.docx]
