# Supplementary material for: Associations between perceived neighborhood environment and physical activity among breast cancer patients engaged in a physical activity program concomitant to cancer treatment: cross-sectional and longitudinal analyses in the DISCO trial (DiscoSpace)
Source: Int J Behav Nutr Phys Act. 2026 Mar 26;23:48. doi: 10.1186/s12966-026-01909-w (PMC13154525; doi:10.1186/s12966-026-01909-w)
Supplement: Supplementary file 4 — Supplementary Material 4. [file 12966_2026_1909_MOESM4_ESM.docx]

**Additional File 4**

| **Characteristics of the 95 non-respondents of the DISCO-SPACE study, France, 2018-2022** | |
| --- | --- |
| **SOCIODEMOGRAPHICS AT BASELINE** |  |
| **Age (years), *mean*** *±* ***SD*** | 50.0 ± 10.4 |
| **Educational level, *n (%)*** |  |
| ≤ baccalaureate | 18 (18.9) |
| 1 to 3 years post-baccalaureate | 9 (9.5) |
| ≥ 4 years post baccalaureate | 7 (7.4) |
| *Missing* | 61 (64.2) |
| **Employment after diagnosis, *n (%)*** |  |
| Active | 7 (7.4) |
| On medical leave or disabled | 24 (25.3) |
| Retired | 4 (4.2) |
| *Missing* | 60 (63.2) |
| **Social deprivation ^a^, *n (%)*** |  |
| Deprived | 20 (21.1) |
| Non-deprived | 61 (64.2) |
| *Missing* | 14 (14.7) |
| **Living situation, *n (%)*** |  |
| Living with a partner | 62 (65.3) |
| Living alone | 19 (20.0) |
| *Missing* | 14 (14.7) |
| **HEALTH AND BEHAVIOUR AT BASELINE** |  |
| **Time since diagnosis (months), *median (IQR)*** | 3.0 (3.0 – 6.5) |
| *Missing, n (%)* | 4 (4.2) |
| **Time since first surgery (months), *median (IQR)*** | 2.0 (1.0 – 2.0) |
| *Missing, n (%)* | 2 (2.1) |
| **BMI (kg/m²), *mean*** *±* ***SD*** | 26.9 ± 5.5 |
| *Missing, n (%)* | 4 (4.2) |
| **BMI categorical, *n (%)*** |  |
| Normal weight | 38 (40.0) |
| Overweight | 28 (29.5) |
| Obesity | 25 (26.3) |
| *Missing* | 4 (4.2) |
| **Quality of life (/100) ^b^, *median (IQR)*** | 66.7 (50 – 83.3) |
| *Missing, n (%)* | 16 (16.8) |
| **Health status (/100) ^c^, *median (IQR)*** | 70.0 (51.3 – 80.0) |
| *Missing, n (%)* | 13 (13.7) |
| **Menopausal status** |  |
| Premenopausal or perimenopausal | 59 (62.1) |
| Postmenopausal | 32 (33.7) |
| *Missing* | 4 (4.2) |
| **Comorbidities, *n (%)*** |  |
| Past or present | 60 (60.3) |
| None | 33 (34.7) |
| *Missing* | 2 (2.1) |
| **INTERVENTION SPECIFICITIES** |  |
| **Trial arm, *n (%)*** |  |
| (A) Individualized, semi-supervised exercise program physical activity program carried out autonomously with a connected device | 21 (22.1) |
| (B) Therapeutic patient education sessions on physical activity | 23 (24.2) |
| (C) Both interventions | 26 (27.4) |
| (D) Control group receiving usual care | 25 (26.3) |
| **COVID-19 pandemic trial status, *n (%)*** |  |
| Before the first national lockdown | 52 (54.7) |
| After the first national lockdown | 43 (45.3) |
| **Received therapies during the intervention (yes), *n (%)*** |  |
| Radiotherapy | 87 (91.6) |
| Hormonotherapy | 75 (78.9) |
| Chemotherapy | 60 (63.2) |
| Immunotherapy | 15 (15.8) |
| **Physical activity measurements** |  |
| **Self-reported physical activity (hour/week) ^d^, median (IQR)** |  |
| At baseline | 3.6 (1.5 – 8.3) |
| At 6-months | 7.0 (3.8 – 15.0) |
| *Missing, n (%)* | 58 (61.1) |
| **6MWD (meters/6min) ^e^, mean** ± **SD** |  |
| At baseline | 555.0 ± 71.3 |
| *Missing, n (%)* | 5 (5.3) |
| At 6-months | 551.0 ± 126.6 |
| *Missing, n (%)* | 64 (67.4) |
| **OBSERVED CHANGES DURING INTERVENTION** |  |
| **Change in BMI ^f^, *n (%)*** |  |
| Weight gain | 3 (3.2) |
| None | 28 (29.5) |
| Weight loss | 2 (2.1) |
| *Missing* | 62 (65.3) |
| **Change in Health status ^g^, *n (%)*** |  |
| Improvement | 7 (7.4) |
| None | 14 (14.7) |
| Deterioration | 11 (11.6) |
| *Missing* | 62 (65.3) |
| **Change in quality of life ^h^, *n (%)*** |  |
| Improvement | 9 (9.5) |
| None | 9 (9.5) |
| Deterioration | 11 (11.6) |
| *Missing* | 66 (69.5) |
| Abbreviations*: IQR* Inter-Quartile Range, *SD* Standard Deviation ; ^a^Assessed using the Evaluation of Deprivation and Inequalities in Health Examination Centers (EPICES) index, with a cut-off score of 30.17 to define social deprivation ; ^b^score calculated from the EORTC QLQ-C30 questionnaire ; ^c^score calculated from the EQ-5D-5L questionnaire ; ^d^Collected with the Recent Physical Activity Questionnaire (RPAQ) ; ^e^Measured by the 6-Minute Walk Test (6MWT) ; ^f^Defined as a 5% increase or decrease over the 6 months ; ^g^Defined as a change of ±8.6 units over the 6 months ; ^h^Defined as a change of ±5 units over the 6 months. | |
