# Supplementary material for: Associations between perceived neighborhood environment and physical activity among breast cancer patients engaged in a physical activity program concomitant to cancer treatment: cross-sectional and longitudinal analyses in the DISCO trial (DiscoSpace)
Source: Int J Behav Nutr Phys Act. 2026 Mar 26;23:48. doi: 10.1186/s12966-026-01909-w (PMC13154525; doi:10.1186/s12966-026-01909-w)
Supplement: Supplementary file 5 — Supplementary Material 5. [file 12966_2026_1909_MOESM5_ESM.docx]

**Additional File 5 –** Summary of the perceived neighborhood environment scores at baseline and interpretation

| **Perceived neighborhood environment scores at baseline (calculated from the ALPHA questionnaire), DISCO-SPACE Study, France, 2018-2022 (n=313)** | | | | | | | |
| --- | --- | --- | --- | --- | --- | --- | --- |
|  | **Crude score** | |  | **Z-score ^b^** | |  | **Missing,**  **n (%)** |
| **Perceived neighborhood environment scores (/crude score scale) *^a^*** | **Median** | **IQR** |  | **Median** | **IQR** |  |  |
| Residential density (/315) | 140.0 | 89.0 – 225.0 |  | -0,234 | -0.968 – 0.990 |  | 33 (10.5) |
| Distance to local facilities (/40) | 22.0 | 17.0 – 30.0 |  | -0,177 | -0.766 – 0.766 |  | 34 (10.9) |
| Cycling infrastructures (/10) | 4.0 | 2.0 – 7.0 |  | -0,199 | -1.028 – 1.045 |  |  |
| Walking infrastructures (/10) | 5.0 | 4.0 – 8.0 |  | -0,221 | -0.713 – 1.253 |  |  |
| Total infrastructures (/20) | 10.0 | 7.0 – 13.0 |  | 0,018 | -0.743 – 0.779 |  |  |
| Safety from crime (/15) | 11.0 | 9.0 – 12.0 |  | 0,455 | -0.637 – 1.002 |  |  |
| Safety from traffic (/15) | 10.0 | 8.0 – 12.0 |  | 0,190 | -0.746 – 1.126 |  |  |
| Total safety (/30) | 20.0 | 18.0 – 22.0 |  | 0,070 | -0.513 – 0.653 |  |  |
| Esthetics (/15) | 10.0 | 9.0 – 11.0 |  | 0,096 | -0.493 – 0.685 |  | 1 (0.3) |
| Pleasure (/20) | 13.0 | 12.0 – 15.0 |  | -0,069 | -0.517 – 0.828 |  | 1 (0.3) |
| Connectivity (/15) | 9.0 | 7.0 – 10.0 |  | 0,277 | -0.660 – 0.745 |  |  |
| Walking and cycling network (/20) | 11.0 | 9.0 – 12.0 |  | 0,152 | -0.590 – 0.523 |  |  |
| Home environment (/6) | 3.0 | 2.0 – 4.0 |  | 0,157 | -0.566 – 0.881 |  |  |
| Abbreviations: *IQR* Inter-Quartile Range*; SD* Standard Deviation ; ^a^ Score calculated from the ALPHA questionnaire (for Assessing Levels of PHysical Activity and Fitness at population level) ; ^b^ Values obtained after standardization (Z-score normalization). | | | | | | | |

Some characteristics showed a relatively concentrated distribution around the median – such as safety from crime (median: 11.0, IQR: 9.0-12.0), esthetics (median: 10.0, IQR: 9.0-11.0), or cycling and walking network (median: 11.0, IQR: 9.0-12.0)), suggesting a homogeneous perception among women. In contrast, distance to local facilities and cycling infrastructure show greater variability, with IQRs of 17.0-30.0 and 2.0-7.0, respectively, which may reflect more contrasting perceptions of these aspects of the neighborhood.
