# Supplementary material for: Associations between perceived neighborhood environment and physical activity among breast cancer patients engaged in a physical activity program concomitant to cancer treatment: cross-sectional and longitudinal analyses in the DISCO trial (DiscoSpace)
Source: Int J Behav Nutr Phys Act. 2026 Mar 26;23:48. doi: 10.1186/s12966-026-01909-w (PMC13154525; doi:10.1186/s12966-026-01909-w)
Supplement: Supplementary file 6 — Supplementary Material 6. [file 12966_2026_1909_MOESM6_ESM.docx]

**Additional File 6**

| **Effect of time on physical activity in analyses assessing the association between perceived neighborhood environment and physical activity, DISCO-SPACE study, France, 2018-2022 (n=313)** | | | | | | | |
| --- | --- | --- | --- | --- | --- | --- | --- |
|  | **Physical Activity Outcome** | | | | | | |
| **Perceived neighborhood environment ^a^** | **Self-reported physical activity ^b^** | | |  | **6MWD ^c^** | | |
|  | **β ^d^** | **95% CI** | **p-value** |  | **β ^d^** | **95% CI** | **p-value** |
| **Residential density** | 0.932 | (0.738 ; 1.126) | <0.001 |  | 23.228 | (14.388 ; 32.067) | <0.001 |
| **Distance to local facilities** | 0.900 | (0.701 ; 1.098) | <0.001 |  | 26.601 | (18.035 ; 35.166) | <0.001 |
| **Cycling infrastructures** | 0.961 | (0.769 ; 1.154) | <0.001 |  | 24.481 | (16.281 ; 32.680) | <0.001 |
| **Walking infrastructures** | 0.963 | (0.771 ; 1.156) | <0.001 |  | 24.353 | (16.149 ; 32.557) | <0.001 |
| **Total infrastructures** | 0.962 | (0.771 ; 1.154) | <0.001 |  | 24.440 | (16.241 ; 32.639) | <0.001 |
| **Safety from crime** | 0.962 | (0.770 ; 1.155) | <0.001 |  | 24.299 | (16.111 ; 32.487) | <0.001 |
| **Safety from traffic** | 0.964 | (0.771 ; 1.156) | <0.001 |  | 24.136 | (15.984 ; 32.288) | <0.001 |
| **Total safety** | 0.963 | (0.771 ; 1.155) | <0.001 |  | 24.149 | (15.988 ; 32.310) | <0.001 |
| **Esthetics** | 0.963 | (0.770 ; 1.155) | <0.001 |  | 24.373 | (16.205 ; 32.540) | <0.001 |
| **Pleasure** | 0.961 | (0.769 ; 1.154) | <0.001 |  | 24.434 | (16.265 ; 32.602) | <0.001 |
| **Connectivity** | 0.962 | (0.769 ; 1.155) | <0.001 |  | 24.452 | (16.265 ; 32.639) | <0.001 |
| **Walking and cycling network** | 0.962 | (0.770 ; 1.155) | <0.001 |  | 24.499 | (16.303 ; 32.694) | <0.001 |
| ^a^ Environmental scores were calculated from the ALPHA questionnaire (for Assessing Levels of PHysical Activity and Fitness at population level) ; ^b^ Self-reported physical activity was calculated from the Recent Physical Activity Questionnaire (RPAQ). The average difference in the outcome self-reported physical activity is expressed by the square root ; ^c^ 6MWD was measured by the 6-Minute Walk Test (6MWT). The average difference in the outcome 6MWD is expressed without transformation ; ^d^ β indicate the overall longitudinal difference in the outcome score using linear mixed models between baseline and 6-month follow-up. This association was estimated by the intervention timepoint term. Analyses were adjusted on: age, social deprivation, educational level, employment status after diagnosis, comorbidities, living with a partner, trial arm, municipality class (except for Residential density score analyses), perceived home environment, COVID-19 pandemic trial status, longitudinal BMI, longitudinal quality of life, longitudinal health status, and corresponding environmental score. | | | | | | | |
