# Supplementary material for: Associations between perceived neighborhood environment and physical activity among breast cancer patients engaged in a physical activity program concomitant to cancer treatment: cross-sectional and longitudinal analyses in the DISCO trial (DiscoSpace)
Source: Int J Behav Nutr Phys Act. 2026 Mar 26;23:48. doi: 10.1186/s12966-026-01909-w (PMC13154525; doi:10.1186/s12966-026-01909-w)
Supplement: Supplementary file 7 — Supplementary Material 7. [file 12966_2026_1909_MOESM7_ESM.docx]

**Additional File 7**

**Statistically significant interactions, and corresponding stratified analyses assessing the association between perceived neighborhood environment and physical activity, DISCO-SPACE study, France, 2018-2022 (n=313)**

| **BMI AT BASELINE** | | | | | | | | |
| --- | --- | --- | --- | --- | --- | --- | --- | --- |
|  | **Self-reported physical activity ^b^** | | | | | | | |
| **Perceived neighborhood environment ^a^** | **< 25kg/m^2^ (n=164)** | | |  | ≥ **25kg/m^2^ (n=148)** | | | **p-int ^g^** |
|  | **β ^d^** | **95% CI** | **p-value** |  | **β ^d^** | **95% CI** | **p-value** |  |
| **Safety from traffic** |  |  |  |  |  |  |  |  |
| Cross-sectional ^e^ | -0.059 | (-0.272;0.153) | 0.584 |  | 0.073 | (-0.129;0.276) | 0.476 | 0.672 |
| Longitudinal ^f^ | **0.340** | **(0.069;0.611)** | **0.014** |  | -0.044 | (-0.302;0.215) | 0.741 | **0.041** |

| **EMPLOYMENT STATUS AFTER DIAGNOSIS** | | | | | | | | | | | |  |
| --- | --- | --- | --- | --- | --- | --- | --- | --- | --- | --- | --- | --- |
|  | **Self-reported physical activity ^b^** | | | | | | | | | | |  |
| **Perceived neighborhood environment** | **Active (n=68)** | | | **On medical leave/ disabled (n=150)** | | | **Retired (n=55)** | | | **p-int ^g^** | |  |
|  | **β ^d^** | **95% CI** | **p-value** | **β ^d^** | **95% CI** | **p-value** | **β ^d^** | **95% CI** | **p-value** | |  | |
| **Safety from traffic** |  |  |  |  |  |  |  |  |  | |  | |
| Cross-sectional ^e^ | 0.126 | (-0.182;0.434) | 0.421 | -0.139 | (-0.335;0.057) | 0.057 | 0.025 | (-0.103;0.552) | 0.178 | | **0.025** | |
| Longitudinal ^f^ | -0.085 | (-0.479;0.309) | 0.670 | 0.181 | (-0.072;0.434) | 0.160 | 0.271 | (-0.150;0.691) | 0.207 | | 0.424 | |
| **Total safety** |  |  |  |  |  |  |  |  |  | |  | |
| Cross-sectional ^e^ | 0.114 | (-0.198;0.425) | 0.473 | 0.130 | (-0.188;0.449) | 0.422 | -0.160 | (-0.364;0.045) | 0.125 | | **0.049** | |
| Longitudinal ^f^ | -0.127 | (-0.521;0.267) | 0.527 | 0.322 | (-0.081;0.725) | 0.117 | 0.186 | (-0.071;0.443) | 0.157 | | 0.265 | |
|  | **6MWD ^c^** | | | | | | | | | | |  |
| **Perceived neighborhood environment** | **Active(n=68)** | | | **On medical leave/ disabled (n=150)** | | | **Retired (n=55)** | | | **p-int ^g^** | |  |
|  | **β ^d^** | **95% CI** | **p-value** | **β ^d^** | **95% CI** | **p-**  **value** | **β ^d^** | **95% CI** | **p-value** | |  | |
| **Total infrastructures** |  |  |  |  |  |  |  |  |  | |  | |
| Cross-sectional ^e^ | -11.172 | (-5.975;28.318) | 0.201 | -4.736 | (-15.808;6.337) | 0.401 | **15.291** | **(0.118;30.465)** | **0.048** | | **0.049** | |
| Longitudinal ^f^ | -10.971 | (-27.779;5.836) | 0.200 | 3.658 | (-6.989;14.305) | 0.500 | 3.935 | (-11.094;18.964) | 0.607 | | 0.312 | |

| **AGE AT BASELINE** | | | | | | | | |
| --- | --- | --- | --- | --- | --- | --- | --- | --- |
|  | **6MWD ^c^** | | | | | | | |
| **Perceived neighborhood environment** | **< 51 years old (n=156)** | | |  | ≥ **51 years old (n=157)** | | | **p-int ^g^** |
|  | **β ^d^** | **95% CI** | **p-value** |  | **β ^d^** | **95% CI** | **p-value** |  |
| **Cycling infrastructures** |  |  |  |  |  |  |  |  |
| Cross-sectional ^e^ | -1.805 | (-13.072;9.462) | 0.753 |  | 11.352 | (-0.246;22.950) | 0.055 | **0.040** |
| Longitudinal ^f^ | -0.612 | (-11.665;10.442) | 0.913 |  | 1.977 | (-9.216;13.170) | 0.729 | 0.742 |
| **Total infrastructures** |  |  |  |  |  |  |  |  |
| Cross-sectional ^e^ | -1.905 | (-13.379;9.568) | 0.744 |  | 9.660 | (-1.796;21.117) | 0.098 | **0.048** |
| Longitudinal ^f^ | -1.866 | (-13.043;9.311) | 0.743 |  | 3.370 | (-7.606;14.347) | 0.547 | 0.505 |
| **Connectivity** |  |  |  |  |  |  |  |  |
| Cross-sectional ^e^ | -1.857 | (-12.423;8.709) | 0.730 |  | 10.685 | (-0.314;21.683) | 0.057 | **0.039** |
| Longitudinal ^f^ | -6.164 | (-17.225;4.897) | 0.274 |  | 0.740 | (-11.462;12.943) | 0.905 | 0.398 |
| **Walking and cycling network** |  |  |  |  |  |  |  |  |
| Cross-sectional ^e^ | -6.458 | (-17.254;4.338) | 0.240 |  | **11.453** | **(0.283;22.623)** | **0.044** | **0.009** |
| Longitudinal ^f^ | -4.408 | (-15.632;6.817) | 0.441 |  | -1.128 | (-12.933;10.678) | 0.851 | 0.681 |

| **TIME SINCE THE FIRST BREAST CANCER SURGERY (AT BASELINE)** | | | | | | | | | | |
| --- | --- | --- | --- | --- | --- | --- | --- | --- | --- | --- |
|  | **6MWD ^c^** | | | | | | | | | |
| **Perceived neighborhood environment** | **≤ 1 month (n=156)** | | |  | **> 1 month (n=157)** | | | | **p-int ^g^** | |
|  | **β ^d^** | **95% CI** | **p-value** |  | **β ^d^** | **95% CI** | **p-value** |  | |  |
| **Walking infrastructures** |  |  |  |  |  |  |  |  | |  |
| Cross-sectional ^e^ | 1.062 | (-9.949;12.073) | 0.850 |  | 2.408 | (-8.359;13.175) | 0.661 | 0.535 | |  |
| Longitudinal ^f^ | -7.531 | (-18.294;3.231) | 0.170 |  | 8.836 | (-2.192;19.864) | 0.116 | **0.047** | |  |

| **HEALTH STATUS AT BASELINE** | | | | | | | | | | |
| --- | --- | --- | --- | --- | --- | --- | --- | --- | --- | --- |
|  | **Self-reported physical activity ^b^** | | | | | | | | | |
| **Perceived neighborhood environment** | **≤ 70/100 (n=155)** | | |  | **> 70/100 (n=156)** | | | | **p-int ^g^** | |
|  | **β ^d^** | **95% CI** | **p-value** |  | **β ^d^** | **95% CI** | **p-value** |  | |  |
| **Residential density** |  |  |  |  |  |  |  |  | |  |
| Cross-sectional ^e^ | -0.050 | (-0.280;0.181) | 0.673 |  | 0.122 | (-0.069;0.313) | 0.211 | 0.872 | |  |
| Longitudinal ^f^ | -0.076 | (-0.371;0.219) | 0.614 |  | **-0.475** | **(-0.722;-0.229)** | **<0.001** | **0.039** | |  |

| **QUALITY OF LIFE AT BASELINE** | | | | | | | | | | |
| --- | --- | --- | --- | --- | --- | --- | --- | --- | --- | --- |
|  | **Self-reported physical activity ^b^** | | | | | | | | | |
| **Perceived neighborhood environment** | **≤ 75/100 (n=148)** | | |  | **> 75/100 (n=148)** | | | | **p-int ^g^** | |
|  | **β ^d^** | **95% CI** | **p-value** |  | **β ^d^** | **95% CI** | **p-value** |  | |  |
| **Distance to local facilities** |  |  |  |  |  |  |  |  | |  |
| Cross-sectional ^e^ | -0.216 | (-0.447;0.014) | 0.066 |  | 0.055 | (-0.181;0.292) | 0.645 | **0.049** | |  |
| Longitudinal ^f^ | 0.185 | (-0.097;0.466) | 0.198 |  | 0.107 | (-0.191;0.404) | 0.482 | 0.702 | |  |
| **Cycling infrastructures** |  |  |  |  |  |  |  |  | |  |
| Cross-sectional ^e^ | **0.252** | **(0.032;0.473)** | **0.025** |  | 0.090 | (-0.132;0.311) | 0.427 | **0.042** | |  |
| Longitudinal ^f^ | -0.090 | (-0.367;0.189) | 0.530 |  | -0.219 | (-0.493;0.055) | 0.117 | 0.516 | |  |
| **Walking infrastructures** |  |  |  |  |  |  |  |  | |  |
| Cross-sectional ^e^ | **0.243** | **(0.037;0.449)** | **0.021** |  | 0.045 | (-0.172;0.262) | 0.683) | **0.007** | |  |
| Longitudinal ^f^ | -0.078 | (-0.344;0.189) | 0.568 |  | **-0.283** | **(-0.565;-0.002)** | **0.048** | 0.291 | |  |
| **Total infrastructures** |  |  |  |  |  |  |  |  | |  |
| Cross-sectional ^e^ | **0.288** | **(0.071;0.505)** | **0.009** |  | 0.086 | (-0.137;0.308) | 0.450 | **0.008** | |  |
| Longitudinal ^f^ | -0.098 | (-0.368;0.173) | 0.479 |  | **-0.278** | **(-0.555;-0.002)** | **0.049** | 0.358 | |  |
|  | **6MWD ^c^** | | | | | | | | | |
| **Perceived neighborhood environment** | **≤ 75/100 (n=148)** | | |  | **> 75/100 (n=148)** | | | | **p-int ^g^** | |
|  | **β ^d^** | **95% CI** | **p-value** |  | **β ^d^** | **95% CI** | **p-value** |  |  |  |
| **Esthetics** |  |  |  |  |  |  |  |  | |  |
| Cross-sectional ^e^ | 1.727 | (-8.784;12.238) | 0.747 |  | 7.321 | (-5.435;20.076) | 0.260 | 0.106 | |  |
| Longitudinal ^f^ | **-14.550** | **(-25.392;-3.707)** | **0.009** |  | 2.943 | (-10.247;16.132) | 0.661 | **0.045** | |  |
| **Pleasure** |  |  |  |  |  |  |  |  | |  |
| Cross-sectional ^e^ | 5.611 | (-5;076;16.298) | 0.303 |  | 8.267 | (-4.222;20.755) | 0.194 | 0.214 | |  |
| Longitudinal ^f^ | **-15.367** | **(-26.575;-4.159)** | **0.007** |  | 2.872 | (-9.965;15.708) | 0.660 | **0.035** | |  |

| **LIVING WITH A PARTNER AT BASELINE** | | | | | | | | | | |
| --- | --- | --- | --- | --- | --- | --- | --- | --- | --- | --- |
|  | **6MWD ^c^** | | | | | | | | | |
| **Perceived neighborhood environment** | **Living alone (n=66)** | | |  | **Living with a partner (n=245)** | | | | **p-int ^g^** | |
|  | **β ^d^** | **95% CI** | **p-value** |  | **β ^d^** | **95% CI** | **p-value** |  | |  |
| **Safety from crime** |  |  |  |  |  |  |  |  | |  |
| Cross-sectional ^e^ | -9.732 | (-25.135;5.671) | 0.215 |  | 1.677 | (-7.722;11.075) | 0.726 | 0.522 | |  |
| Longitudinal ^f^ | 11.805 | (-5.782;29.391) | 0.188 |  | -7.768 | (-16.514;0.978) | 0.082 | **0.044** | |  |
| **Connectivity** |  |  |  |  |  |  |  |  | |  |
| Cross-sectional ^e^ | **17.886** | **(0.479;35.292)** | **0.044** |  | 1.663 | (-6.656;9.982) | 0.695 | **0.019** | |  |
| Longitudinal ^f^ | 3.952 | (-15.200;23.101) | 0.685 |  | -5.433 | (-14.510;3.644) | 0.240 | 0.369 | |  |
| **Walking and cycling network** |  |  |  |  |  |  |  |  | |  |
| Cross-sectional ^e^ | **16.582** | **(0.294;32.869)** | **0.046** |  | -0.985 | (-9.645;7.674) | 0.823 | **0.013** | |  |
| Longitudinal ^f^ | 1.629 | (-16.505;19.764) | 0.860 |  | -4.634 | (-13.726;4.458) | 0.317 | 0.531 | |  |

| **TRIAL ARM** | | | | | | | | | | | | | |
| --- | --- | --- | --- | --- | --- | --- | --- | --- | --- | --- | --- | --- | --- |
|  | **Self-reported physical activity ^b^** | | | | | | | | | | | | |
| **Perceived neighborhood environment** | **(A) Autonomous program with connected device (n=79)** | | | **(B) Therapeutic patient education (n=76)** | | | **(C) Both interventions (n=77)** | | | **(D) Control (n=81)** | | | **p-int ^g^** |
|  | **β ^d^** | **95% CI** | **p-**  **value** | **β ^d^** | **95% CI** | **p-value** | **β^c^** | **95% CI** | **p-value** | **β ^d^** | **95% CI** | **p-value** |  |
| **Esthetics** |  |  |  |  |  |  |  |  |  |  |  |  |  |
| Cross-sectional ^e^ | 0.122 | (-0.192;0.437) | 0.446 | 0.058 | (-0.237;0.352) | 0.701 | -0.152 | (-0.430;0.127) | 0.285 | 0.072 | (0.214;0.358) | 0.632 | **0.016** |
| Longitudinal ^f^ | -0.135 | (-0.532;0.262) | 0.505 | 0.363 | (-0.020;0.746) | 0.063 | **-0.659** | **(-1.195;0.123)** | **0.016** | -0.167 | (-0.531;0.196) | 0.366 | 0.504 |
|  | **6MWD ^c^** | | | | | | | | | | | | |
| **Perceived neighborhood environment** | **(A) Autonomous physical activity program with connected device (n=79)** | | | **(B) Therapeutic patient education (n=76)** | | | **(C) Both interventions (n=77)** | | | **(D) Control (n=81)** | | | **p-int ^g^** |
|  | **β ^d^** | **95% CI** | **p-**  **value** | **β ^d^** | **95% CI** | **p-value** | **β ^d^** | **95% CI** | **p-value** | **β ^d^** | **95% CI** | **p-value** |  |
| **Distance to local facilities** |  |  |  |  |  |  |  |  |  |  |  |  |  |
| Cross-sectional ^e^ | -6.702 | (-23.534;10.130) | 0.434 | -10.201 | (-26.083;5.662) | 0.207 | 2.393 | (-14.526;19.312) | 0.781 | **-28.237** | **(-43.733;-12.740)** | **<0.001** | **0.019** |
| Longitudinal ^f^ | -0.940 | (-19.202;17.323) | 0.919 | 13.006 | (-4.341;30.354) | 0.141 | -8.166 | (-26.321;9.989) | 0.377 | -1.543 | (-17.411;14.326) | 0.849 | 0.379 |
| **Connectivity** |  |  |  |  |  |  |  |  |  |  |  |  |  |
| Cross-sectional ^e^ | 2.561 | (-12.408;17.531) | 0.737 | 14.666 | (-0.130;29.462) | 0.052 | -0.010 | (-13.828;13.801) | 0.999 | 0.911 | (-15.007;16.828) | 0.911 | 0.741 |
| Longitudinal ^f^ | 8.918 | (-6.970;24.805) | 0.271 | **-23.484** | **(-39.518;-7.449)** | **0.004** | -6.220 | (-21.552;9.112) | 0.426 | 8.885 | (-9.500;27.266) | 0.343 | **0.015** |
| **Walking and cycling network** |  |  |  |  |  |  |  |  |  |  |  |  |  |
| Cross-sectional ^e^ | -3.906 | (-18.787;10.975) | 0.606 | 10.564 | (-4.403;25.530) | 0.166 | -0.038 | (-14.144;14.067) | 0.996 | 4.729 | (-11.235;20.693) | 0.561 | 0.905 |
| Longitudinal ^f^ | 12.972 | (-2.974;28.915) | 0.111 | **-20.845** | **(-36.747;-4.944)** | **0.010** | -4.890 | (-20.520;10.740) | 0.539 | -0.005 | (-17.376;17.366) | 0.999 | **0.027** |

| Abbrevations: *6MWD* 6-minute Walk Distance; *p-int* p for interaction ; Values in bold are statistically significant (P <0.05) ; ^a^ Environmental scores were calculated from the ALPHA questionnaire (for Assessing Levels of PHysical Activity and Fitness at population level) ; ^b^ Self-reported physical activity was calculated from the Recent Physical Activity Questionnaire (RPAQ). The average difference in the outcome self-reported physical activity is expressed by the square root ; ^c^ 6MWD was measured by the 6-Minute Walk Test (6MWT). The average difference in the outcome 6MWD is expressed without transformation ; ^d^ The β indicate the overall longitudinal difference in the outcome score using linear mixed models per 1 SD of perceived built environment score after a standardized Z-score transformation. Analyses were adjusted on: age, social deprivation, educational level, employment status after diagnosis, comorbidities, living with a partner, trial arm, municipality class (except for Residential density score analyses), perceived home environment, COVID-19 pandemic trial status, longitudinal BMI, longitudinal quality of life, and longitudinal health status. Stratified factors were excluded from adjustment ; ^e^ The cross-sectional association of perceived neighborhood environment and physical activity is estimated by the environmental perception score term ; ^f^ The longitudinal association of perceived neighborhood environment and physical activity is estimated by the interaction term between the intervention visit and the environmental perception score ; ^g^ For cross-sectional associations, two-way ANOVA was performed to test the interaction between the effect modifier and the explanatory variable; for longitudinal associations, three-way ANOVA was performed to test the interaction between the effect modifier, the explanatory variable, and the timepoint. |
| --- |
