# Supplementary material for: Associations between perceived neighborhood environment and physical activity among breast cancer patients engaged in a physical activity program concomitant to cancer treatment: cross-sectional and longitudinal analyses in the DISCO trial (DiscoSpace)
Source: Int J Behav Nutr Phys Act. 2026 Mar 26;23:48. doi: 10.1186/s12966-026-01909-w (PMC13154525; doi:10.1186/s12966-026-01909-w)
Supplement: Supplementary file 8 — Supplementary Material 8. [file 12966_2026_1909_MOESM8_ESM.docx]

**Additional File 8**

| **Sensitivity analysis adjusted for the completion period of the ALPHA environmental questionnaire, assessing the association between perceived neighborhood environment and physical activity, DISCO-SPACE study, France, 2018–2022 (n=313)** | | | | | | | |
| --- | --- | --- | --- | --- | --- | --- | --- |
| **Perceived neighborhood environment ^a^** | **Self-reported physical activity ^b^** | | | | | | |
|  | **Main analysis** | | |  | **Sensitivity analysis** | | |
|  | **β ^d^** | **95% CI** | **p-value** |  | **β ^d^** | **95% CI** | **p-value** |
| **Residential density** |  |  |  |  |  |  |  |
| Cross-sectional ^e^ | 0.047 | (-0.105 ; 0.199) | 0.542 |  | 0.053 | (-0.099 ; 0.206) | 0.492 |
| Longitudinal ^f^ | **-0.306** | **(-0.494 ; -0.117)** | **0.002** |  | **-0.309** | **(-0.497 ; -0.120)** | **0.001** |
| **Distance to local facilities** |  |  |  |  |  |  |  |
| Cross-sectional ^e^ | -0.071 | (-0.242 ; 0.100) | 0.413 |  | -0.082 | (-0.255 ; 0.091) | 0.351 |
| Longitudinal ^f^ | 0.181 | (-0.014 ; 0.377) | 0.069 |  | 0.177 | **(**-0.017 **;** 0.372**)** | 0.073 |
| **Cycling infrastructures** |  |  |  |  |  |  |  |
| Cross-sectional ^e^ | **0.210** | **(0.048 ; 0.372)** | **0.011** |  | **0.214** | **(0.051 ; 0.377)** | **0.010** |
| Longitudinal ^f^ | -0.151 | (-0.340 ; 0.038) | 0.117 |  | -0.150 | (-0.339 ; 0.039) | 0.119 |
| **Walking infrastructures** |  |  |  |  |  |  |  |
| Cross-sectional ^e^ | **0.170** | **(0.016 ; 0.324)** | **0.031** |  | **0.176** | **(0.020 ; 0.332)** | **0.027** |
| Longitudinal ^f^ | -0.159 | (-0.348 ; 0.030) | 0.099 |  | -0.158 | (-0.347 ; 0.030) | 0.099 |
| **Total infrastructures** |  |  |  |  |  |  |  |
| Cross-sectional ^e^ | **0.226** | **(0.063 ; 0.388)** | **0.007** |  | **0.232** | **(0.068 ; 0.395)** | **0.006** |
| Longitudinal ^f^ | -0.175 | (-0.363 ; 0.013) | 0.068 |  | -0.174 | (-0.361 ; 0.014) | 0.069 |
| **Safety from crime** |  |  |  |  |  |  |  |
| Cross-sectional ^e^ | -0.076 | (-0.235 ; 0.083) | 0.347 |  | -0.082 | (-0.242 ; 0.078) | 0.315 |
| Longitudinal ^f^ | 0.113 | (-0.075 ; 0.301) | 0.240 |  | 0.113 | (-0.074 ; 0.301) | 0.235 |
| **Safety from traffic** |  |  |  |  |  |  |  |
| Cross-sectional ^e^ | -0.009 | (-0.155 ; 0.138) | 0.906 |  | -0.012 | (-0.159 ; 0.135) | 0.870 |
| Longitudinal ^f^ | 0.138 | (-0.049 ; 0.326) | 0.148 |  | 0.140 | (-0.047 ; 0.327) | 0.143 |
| **Total safety** |  |  |  |  |  |  |  |
| Cross-sectional ^e^ | -0.040 | (-0.193 ; 0.114) | 0.610 |  | -0.045 | (-0.199 ; 0.109) | 0.567 |
| Longitudinal ^f^ | 0.147 | (-0.041 ; 0.335) | 0.125 |  | 0.148 | (-0.039 ; 0.336) | 0.121 |
| **Esthetics** |  |  |  |  |  |  |  |
| Cross-sectional ^e^ | 0.017 | (-0.139 ; 0.173) | 0.828 |  | 0.016 | (-0.140 ; 0.173) | 0.837 |
| Longitudinal ^f^ | 0.152 | (-0.037 ; 0.340) | 0.114 |  | 0.152 | (-0.036 ; 0.340) | 0.114 |
| **Pleasure** |  |  |  |  |  |  |  |
| Cross-sectional ^e^ | 0.032 | (-0.125 ; 0.188) | 0.692 |  | 0.031 | (-0.125 ; 0.188) | 0.694 |
| Longitudinal ^f^ | 0.174 | (-0.015 ; 0.364) | 0.071 |  | 0.174 | (-0.014 ; 0.363) | 0.070 |
| **Connectivity** |  |  |  |  |  |  |  |
| Cross-sectional ^e^ | 0.120 | (-0.030 ; 0.269) | 0.118 |  | 0.129 | (-0.022 ; 0.280) | 0.093 |
| Longitudinal ^f^ | -0.049 | (-0.241 ; 0.144) | 0.620 |  | -0.052 | (-0.244 ; 0.139) | 0.591 |
| **Walking and cycling network** |  |  |  |  |  |  |  |
| Cross-sectional ^e^ | **0.161** | **(0.008 ; 0.314)** | **0.039** |  | **0.170** | **(0.016 ; 0.324)** | **0.030** |
| Longitudinal ^f^ | -0.140 | (-0.331 ; 0.050) | 0.148 |  | -0.145 | (-0.335 ; 0.134) | 0.134 |
| **Perceived neighborhood environment ^a^** | **6MWD ^c^** | | | | | | |
|  | **Main analysis** | | |  | **Sensitivity analysis** | | |
|  | **β ^d^** | **95% CI** | **p-value** |  | **β ^d^** | **95% CI** | **p-value** |
| **Residential density** |  |  |  |  |  |  |  |
| Cross-sectional ^e^ | 0.189 | (-8.377 ; 8.755) | 0.965 |  | -0.626 | (-9.173 ; 7.921) | 0.886 |
| Longitudinal ^f^ | 2.307 | (-6.163 ; 10.776) | 0.593 |  | 2.423 | (-6.029 ; 10.876) | 0.573 |
| **Distance to local facilities** |  |  |  |  |  |  |  |
| Cross-sectional ^e^ | **-11.363** | **(-20.607 ; -2.118)** | **0.016** |  | **-12.079** | **(-21.395 ; -2.762)** | **0.011** |
| Longitudinal ^f^ | 0.160 | (-8.379 ; 8.700) | 0.971 |  | 0.355 | (-8.173 ; 8.883) | 0.935 |
| **Cycling infrastructures** |  |  |  |  |  |  |  |
| Cross-sectional ^e^ | 4.509 | (-4.081 ; 13.098) | 0.303 |  | 3.943 | (-4.615 ; 12.501) | 0.366 |
| Longitudinal ^f^ | 0.058 | (-7.725 ; 7.841) | 0.988 |  | -0.013 | (-7.791 ; 7.766) | 0.997 |
| **Walking infrastructures** |  |  |  |  |  |  |  |
| Cross-sectional ^e^ | 1.685 | (-6.376 ; 9.747) | 0.681 |  | 1.627 | (-6.458 ; 9.712) | 0.693 |
| Longitudinal ^f^ | 0.815 | (-7.023 ; 8.653) | 0.838 |  | 0.694 | (-7.142 ; 8.529) | 0.862 |
| **Total infrastructures** |  |  |  |  |  |  |  |
| Cross-sectional ^e^ | 3.837 | (-4.806 ; 12.479) | 0.384 |  | 3.454 | (-5.178 ; 12.085) | 0.432 |
| Longitudinal ^f^ | 0.988 | (-7.338 ; 8.208) | 0.912 |  | 0.332 | (-7.436 ; 8.101) | 0.933 |
| **Safety from crime** |  |  |  |  |  |  |  |
| Cross-sectional ^e^ | -0.979 | (-9.288 ; 7.330) | 0.817 |  | -1.385 | (-9.650 ; 6.881) | 0.742 |
| Longitudinal ^f^ | -3.964 | (-11.802 ; 3.874) | 0.321 |  | -4.199 | (-12.037 ; 3.640) | 0.293 |
| **Safety from traffic** |  |  |  |  |  |  |  |
| Cross-sectional ^e^ | 5.344 | (-2.126 ; 12.815) | 0.160 |  | 5.222 | (-2.221 ; 12.666) | 0.169 |
| Longitudinal ^f^ | -6.353 | (-14.044 ; 1.338) | 0.105 |  | -6.627 | (-14.310 ; 1.055) | 0.091 |
| **Total safety** |  |  |  |  |  |  |  |
| Cross-sectional ^e^ | 3.224 | (-4.727 ; 11.175) | 0.426 |  | 2.937 | (-4.975 ; 10.850) | 0.466 |
| Longitudinal ^f^ | -6.114 | (-13.901 ; 1.673) | 0.124 |  | -6.414 | (-14.196 ; 1.368) | 0.106 |
| **Esthetics** |  |  |  |  |  |  |  |
| Cross-sectional ^e^ | 4.879 | (-4.252 ; 12.010) | 0.349 |  | 4.185 | (-3.905 ; 12.274) | 0.310 |
| Longitudinal ^f^ | -7.150 | (-15.223 ; 0.923) | 0.082 |  | -7.076 | (-15.143 ; 0.992) | 0.085 |
| **Pleasure** |  |  |  |  |  |  |  |
| Cross-sectional ^e^ | 6.207 | (-1.959 ; 14.373) | 0.136 |  | 6.895 | (-1.230 ; 15.021) | 0.096 |
| Longitudinal ^f^ | -6.862 | (-15.052 ; 1.328) | 0.100 |  | -6.862 | (-15.043 ; 1.320) | 0.100 |
| **Connectivity** |  |  |  |  |  |  |  |
| Cross-sectional ^e^ | 4.457 | (-3.252 ; 12.166) | 0.257 |  | 5.455 | (-2.269 ; 13.180) | 0.166 |
| Longitudinal ^f^ | -3.482 | (-11.676 ; 4.713) | 0.404 |  | -3.492 | (-11.683 ; 4.699) | 0.403 |
| **Walking and cycling network** |  |  |  |  |  |  |  |
| Cross-sectional ^e^ | 2.630 | (-5.290 ; 10.551) | 0.514 |  | 3.514 | (-4.409 ; 11.437) | 0.384 |
| Longitudinal ^f^ | -3.191 | (-11.301 ; 4.919) | 0.440 |  | -3.220 | (-11.326 ; 4.885) | 0.435 |
| Values in bold are statistically significant (P <0.05) ; The completion period of the ALPHA environmental questionnaire was modalized as a 3-modality variable depending on the timeline with DISCO intervention: before (n=59)/during (n=69)/after intervention (n=185) ;  ^a^ Environmental scores were calculated from the ALPHA questionnaire (for Assessing Levels of PHysical Activity and Fitness at population level) ; ^b^ Self-reported physical activity was calculated from the Recent Physical Activity Questionnaire (RPAQ). The average difference in the outcome self-reported physical activity is expressed by the square root ; ^c^ 6MWD was measured by the 6-Minute Walk Test (6MWT). The average difference in the outcome 6MWD is expressed without transformation ; ^d^ The β indicate the overall longitudinal difference in the outcome score using linear mixed models per 1 SD of perceived built environment score after a standardized Z-score transformation. Analyses were adjusted on: age, social deprivation, educational level, employment status after diagnosis, comorbidities, living with a partner, trial arm, municipality class (except for Residential density score analyses), perceived home environment, COVID-19 pandemic trial status, longitudinal BMI, longitudinal quality of life, longitudinal health status and completion period of the ALPHA environmental questionnaire ; ^e^ The cross-sectional association of perceived neighborhood environment and physical activity is estimated by the environmental perception score term ; ^f^ The longitudinal association of perceived neighborhood environment and physical activity is estimated by the interaction term between the intervention visit and the environmental perception score. | | | | | | | |
