# Supplementary material for: Associations between perceived neighborhood environment and physical activity among breast cancer patients engaged in a physical activity program concomitant to cancer treatment: cross-sectional and longitudinal analyses in the DISCO trial (DiscoSpace)
Source: Int J Behav Nutr Phys Act. 2026 Mar 26;23:48. doi: 10.1186/s12966-026-01909-w (PMC13154525; doi:10.1186/s12966-026-01909-w)
Supplement: Supplementary file 9 — Supplementary Material 9. [file 12966_2026_1909_MOESM9_ESM.docx]

**Additional File 9**

| **Sensitivity analysis excluding participants reporting self-reported physical activity above 99^th^ percentile (2 participants excluded), assessing the association between perceived neighborhood environment and physical activity, DISCO-SPACE study, France, 2018-2022 (n=313)** | | | | | | | |
| --- | --- | --- | --- | --- | --- | --- | --- |
| **Perceived neighborhood environment ^a^** | **Self-reported physical activity ^b^** | | | | | | |
|  | **Main analysis (n=313)** | | |  | **Sensitivity analysis (n=311)** | | |
|  | **β ^c^** | **95% CI** | **p-value** |  | **β ^c^** | **95% CI** | **p-value** |
| **Residential density** |  |  |  |  |  |  |  |
| Cross-sectional ^d^ | 0.047 | (-0.105 ; 0.199) | 0.542 |  | 0.040 | (-0.106 ; 0.186) | 0.588 |
| Longitudinal ^e^ | **-0.306** | **(-0.494 ; -0.117)** | **0.002** |  | **-0.296** | **(-0.479 ; -0.114)** | **0.002** |
| **Distance to local facilities** |  |  |  |  |  |  |  |
| Cross-sectional ^d^ | -0.071 | (-0.242 ; 0.100) | 0.413 |  | -0.053 | (-0.212 ; 0.106) | 0.510 |
| Longitudinal ^e^ | 0.181 | (-0.014 ; 0.377) | 0.069 |  | **0.204** | **(0.014 ; 0.395)** | **0.036** |
| **Cycling infrastructures** |  |  |  |  |  |  |  |
| Cross-sectional ^d^ | **0.210** | **(0.048 ; 0.372)** | **0.011** |  | **0.238** | **(0.087 ; 0.389)** | **0.002** |
| Longitudinal ^e^ | -0.151 | (-0.340 ; 0.038) | 0.117 |  | **-0.195** | **(-0.378 ; -0.012)** | **0.036** |
| **Walking infrastructures** |  |  |  |  |  |  |  |
| Cross-sectional ^d^ | **0.170** | **(0.016 ; 0.324)** | **0.031** |  | **0.152** | **(0.008 ; 0.296)** | **0.038** |
| Longitudinal ^e^ | -0.159 | (-0.348 ; 0.030) | 0.099 |  | -0.171 | (-0.354 ; 0.012) | 0.067 |
| **Total infrastructures** |  |  |  |  |  |  |  |
| Cross-sectional ^d^ | **0.226** | **(0.063 ; 0.388)** | **0.007** |  | **0.231** | **(0.080 ; 0.382)** | **0.003** |
| Longitudinal ^e^ | -0.175 | (-0.363 ; 0.013) | 0.068 |  | **-0.207** | **(-0.389 ; -0.026)** | **0.025** |
| **Safety from crime** |  |  |  |  |  |  |  |
| Cross-sectional ^d^ | -0.076 | (-0.235 ; 0.083) | 0.347 |  | -0.069 | (-0.217 ; 0.079) | 0.361 |
| Longitudinal ^e^ | 0.113 | (-0.075 ; 0.301) | 0.240 |  | 0.081 | (-0.101 ; 0.262) | 0.384 |
| **Safety from traffic** |  |  |  |  |  |  |  |
| Cross-sectional ^d^ | -0.009 | (-0.155 ; 0.138) | 0.906 |  | -0.011 | (-0.148 ; 0.126) | 0.876 |
| Longitudinal ^e^ | 0.138 | (-0.049 ; 0.326) | 0.148 |  | 0.095 | (-0.086 ; 0.277) | 0.304 |
| **Total safety** |  |  |  |  |  |  |  |
| Cross-sectional ^d^ | -0.040 | (-0.193 ; 0.114) | 0.610 |  | -0.039 | (-0.182 ; 0.104) | 0.594 |
| Longitudinal ^e^ | 0.147 | (-0.041 ; 0.335) | 0.125 |  | 0.103 | (-0.079 ; 0.285) | 0.268 |
| **Esthetics** |  |  |  |  |  |  |  |
| Cross-sectional ^d^ | 0.017 | (-0.139 ; 0.173) | 0.828 |  | -0.007 | (-0.153 ; 0.139) | 0.925 |
| Longitudinal ^e^ | 0.152 | (-0.037 ; 0.340) | 0.114 |  | 0.147 | (-0.035 ; 0.330) | 0.114 |
| **Pleasure** |  |  |  |  |  |  |  |
| Cross-sectional ^d^ | 0.032 | (-0.125 ; 0.188) | 0.692 |  | 0.007 | (-0.139 ; 0.153) | 0.928 |
| Longitudinal ^e^ | 0.174 | (-0.015 ; 0.364) | 0.071 |  | 0.169 | (-0.014 ; 0.352) | 0.071 |
| **Connectivity** |  |  |  |  |  |  |  |
| Cross-sectional ^d^ | 0.120 | (-0.030 ; 0.269) | 0.118 |  | 0.115 | (-0.025 ; 0.255) | 0.108 |
| Longitudinal ^e^ | -0.049 | (-0.241 ; 0.144) | 0.620 |  | -0.111 | (-0.297 ; 0.074) | 0.239 |
| **Walking and cycling network** |  |  |  |  |  |  |  |
| Cross-sectional ^d^ | **0.161** | **(0.008 ; 0.314)** | **0.039** |  | **0.148** | **(0.005 ; 0.291)** | **0.042** |
| Longitudinal ^e^ | -0.140 | (-0.331 ; 0.050) | 0.148 |  | **-0.195** | **(-0.379 ; -0.012)** | **0.037** |
| Values in bold are statistically significant (P <0.05) ; ^a^ Environmental scores were calculated from the ALPHA questionnaire (for Assessing Levels of PHysical Activity and Fitness at population level) ; ^b^ Self-reported physical activity was calculated from the Recent Physical Activity Questionnaire (RPAQ). The average difference in the outcome self-reported physical activity is expressed by the square root ; ^c^ The β indicate the overall longitudinal difference in the outcome score using linear mixed models per 1 SD of perceived built environment score after a standardized Z-score transformation. Analyses were adjusted on: age, social deprivation, educational level, employment status after diagnosis, comorbidities, living with a partner, trial arm, municipality class (except for Residential density score analyses), perceived home environment, COVID-19 pandemic trial status, longitudinal BMI, longitudinal quality of life, and longitudinal health status ; ^d^ The cross-sectional association of perceived neighborhood environment and physical activity is estimated by the environmental perception score term ; ^e^ The longitudinal association of perceived neighborhood environment and physical activity is estimated by the interaction term between the intervention visit and the environmental perception score. | | | | | | | |
