# Supplementary material for: Associations between perceived neighborhood environment and physical activity among breast cancer patients engaged in a physical activity program concomitant to cancer treatment: cross-sectional and longitudinal analyses in the DISCO trial (DiscoSpace)
Source: Int J Behav Nutr Phys Act. 2026 Mar 26;23:48. doi: 10.1186/s12966-026-01909-w (PMC13154525; doi:10.1186/s12966-026-01909-w)
Supplement: Supplementary file 10 — Supplementary Material 10. [file 12966_2026_1909_MOESM10_ESM.docx]

**Additional File 10**

**Sensitivity analysis stratified by cancer treatment subgroups, assessing the association between perceived neighborhood environment and physical activity, DISCO-SPACE study, France, 2018-2022 (n=313)**

|  | | | | | | | | | | | | |
| --- | --- | --- | --- | --- | --- | --- | --- | --- | --- | --- | --- | --- |
|  | **Self-reported physical activity ^b^** | | | | | | | | | | | |
| **Perceived neighborhood environment ^a^** | **Chemotherapy (n=180)** | | | **Hormonotherapy (n=254)** | | | **Immunotherapy (n=44)** | | | **Radiotherapy (n=284)** | | |
|  | **β ^d^** | **95% CI** | **p-value** | **β ^d^** | **95% CI** | **p-value** | **β ^d^** | **95% CI** | **p-value** | **β ^d^** | **95% CI** | **p-value** |
| **Residential density** |  |  |  |  |  |  |  |  |  |  |  |  |
| Cross-sectional^e^ | 0.082 | (-0.132;0.297) | 0.450 | 0.008 | (-0.154;0.170) | 0.920 | -0.091 | (-0.508;0.325) | 0.661 | 0.074 | (-0.080;0.229) | 0.346 |
| Longitudinal^f^ | **-0.361** | **(-0.616**;**-0.107)** | **0.006** | **-0.328** | (**-0.536**;**-0.120**) | **0.002** | -0.409 | (-0.896;0.077) | 0.097 | **-0.301** | (-**0.503;-0.100**) | **0.004** |
| **Distance to local facilities** |  |  |  |  |  |  |  |  |  |  |  |  |
| Cross-sectional^e^ | -0.116 | (-0.371;0.140) | 0.373 | -0.030 | (-0.220;0.159) | 0.754 | 0.224 | (-0.524;0.971) | 0.550 | -0.110 | (-0.287;0.066) | 0.221 |
| Longitudinal^f^ | 0.188 | (-0.076;0.453) | 0.162 | 0.206 | (-0.008;0.421) | 0.060 | 0.032 | (-0.597;0.660) | 0.919 | 0.175 | (-0.034;0.384) | 0.100 |
| **Cycling infrastructures** |  |  |  |  |  |  |  |  |  |  |  |  |
| Cross-sectional^e^ | **0.332** | **(0.120;0.544)** | **0.002** | 0.143 | (-0.036;0.321) | 0.116 | -0.163 | (-0.807;0.480) | 0.613 | **0.196** | **(0.028;0.363)** | **0.022** |
| Longitudinal^f^ | -0.179 | (-0.433;0.075) | 0.167 | -0.138 | (-0.351;0.075) | 0.202 | 0.239 | (-0.312;0.789) | 0.389 | -0.174 | (-0.375;0.027) | 0.089 |
| **Walking infrastructures** |  |  |  |  |  |  |  |  |  |  |  |  |
| Cross-sectional^e^ | **0.261** | **(0.048;0.474)** | **0.016** | 0.123 | (-0.048;0.474) | 0.158 | -0.031 | (-0.571;0.510) | 0.910 | **0.176** | **(0.018;0.335)** | **0.029** |
| Longitudinal^f^ | -0.165 | (-0.420;0.090) | 0.204 | -0.105 | (-0.318;0.108) | 0.334 | -0.018 | (-0.647;0.611) | 0.954 | -0.198 | (-0.397;0.002) | 0.052 |
| **Total infrastructures** |  |  |  |  |  |  |  |  |  |  |  |  |
| Cross-sectional^e^ | **0.369** | **(0.148;0.591)** | **0.001** | 0.157 | (-0.022;0.335) | 0.085 | -0.140 | (-0.788;0.509) | 0.668 | **0.220** | **(0.052;0.388)** | **0.010** |
| Longitudinal^f^ | -0.204 | (-0.460;0.051) | 0.116 | -0.138 | (-0.349;0.073) | 0.199 | 0.173 | (-0.446;0.792) | 0.578 | **-0.210** | **(-0.410;-0.011)** | **0.039** |
| **Safety from crime** |  |  |  |  |  |  |  |  |  |  |  |  |
| Cross-sectional^e^ | -0.111 | (-0.336;0.114) | 0.332 | 0.026 | (-0.150;0.203) | 0.769 | -0.114 | (-0.813;0.585) | 0.745 | -0.084 | (-0.245;0.076) | 0.302 |
| Longitudinal^f^ | 0.125 | (-0.138;0.388) | 0.349 | 0.017 | (-0.196;0.231) | 0.872 | -0.0005 | (-0.692;0.691) | 0.999 | 0.132 | (-0.061;0.325) | 0.181 |
| **Safety from traffic** |  |  |  |  |  |  |  |  |  |  |  |  |
| Cross-sectional^e^ | -0.120 | (-0.328;0.088) | 0.256 | 0.039 | (-0.119;0.198) | 0.626 | -0.146 | (-0.677;0.385) | 0.584 | 0.007 | (-0.143;0.157) | 0.926 |
| Longitudinal^f^ | 0.126 | (-0.130;0.382) | 0.335 | 0.099 | (-0.109;0.307) | 0.351 | -0.188 | (-0.827;0.450) | 0.557 | 0.135 | (-0.060;0.330) | 0.175 |
| **Total safety** |  |  |  |  |  |  |  |  |  |  |  |  |
| Cross-sectional^e^ | -0.132 | (-0.345;0.081) | 0.223 | 0.045 | (-0.124;0.215) | 0.599 | -0.190 | (-0.825;0.446) | 0.553 | -0.034 | (-0.190;0.123) | 0.674 |
| Longitudinal^f^ | 0.142 | (-0.114;0.398) | 0.277 | 0.074 | (-0.139;0.287) | 0.497 | -0.113 | (-0.800;0.574) | 0.743 | 0.155 | (-0.039;0.350) | 0.117 |
| **Esthetics** |  |  |  |  |  |  |  |  |  |  |  |  |
| Cross-sectional^e^ | -0.036 | (-0.269;0.198) | 0.764 | 0.123 | (-0.052;0.298) | 0.168 | 0.150 | (-0.510;0.809) | 0.651 | 0.027 | (-0.132;0.186) | 0.740 |
| Longitudinal^f^ | 0.044 | (-0.229;0.318) | 0.750 | 0.143 | (-0.077;0.362) | 0.203 | 0.020 | (-0.668;0.707) | 0.955 | 0.126 | (-0.070;0.322) | 0.208 |
| **Pleasure** |  |  |  |  |  |  |  |  |  |  |  |  |
| Cross-sectional^e^ | -0.053 | (-0.290;0.184) | 0.659 | 0.128 | (-0.045;0.301) | 0.147 | 0.152 | (-0.467;0.771) | 0.624 | 0.036 | (-0.123;0.195) | 0.655 |
| Longitudinal^f^ | 0.109 | (-0.170;0.388) | 0.443 | 0.157 | (-0.061;0.375) | 0.157 | -0.078 | (-0.740;0.583) | 0.814 | 0.156 | (-0.040;0.352) | 0.119 |
| **Connectivity** |  |  |  |  |  |  |  |  |  |  |  |  |
| Cross-sectional^e^ | 0.123 | (-0.087;0.333) | 0.250 | 0.163 | (-0.0001;0.326) | 0.050 | -0.118 | (-0.737;0.501) | 0.703 | 0.097 | (-0.060;0.253) | 0.225 |
| Longitudinal^f^ | -0.137 | (-0.396;0.112) | 0.300 | -0.028 | (-0.242;0.186) | 0.796 | -0.320 | (-0.880;0.240) | 0.258 | -0.009 | (-0.213;0.195) | 0.929 |
| **Walking and cycling network** |  |  |  |  |  |  |  |  |  |  |  |  |
| Cross-sectional^e^ | 0.141 | (-0.074;0.355) | 0.198 | **0.211** | **(0.041;0.381)** | **0.015** | -0.080 | (-0.650;0.490) | 0.779 | 0.156 | (-0.003;0.315) | 0.054 |
| Longitudinal^f^ | -0.186 | (-0.442;0.070) | 0.155 | -0.130 | (-0.345;0.085) | 0.236 | -0.330 | (-0.871;0.212) | 0.228 | -0.101 | (-0.303;0.100) | 0.324 |
|  | **6MWD ^c^** | | | | | | | | | | | |
| **Perceived neighborhood environment ^a^** | **Chemotherapy (n=180)** | | | **Hormonotherapy (n=254)** | | | **Immunotherapy (n=44)** | | | **Radiotherapy (n=284)** | | |
|  | **β ^d^** | **95% CI** | **p-value** | **β ^d^** | **95% CI** | **p-value** | **β ^d^** | **95% CI** | **p-value** | **β ^d^** | **95% CI** | **p-value** |
| **Residential density** |  |  |  |  |  |  |  |  |  |  |  |  |
| Cross-sectional^e^ | 1.042 | (-11.288;13.373) | 0.868 | 1.192 | (-8.053;10.437) | 0.800 | 17.681 | (-20.071;55.434) | 0.350 | 1.859 | (-7.205;10.923) | 0.687 |
| Longitudinal^f^ | -2.578 | (-15.352;10.196) | 0.691 | -1.066 | (-10.321;8.190) | 0.821 | -5.620 | (-33.540;22.300) | 0.687 | 0.916 | (-8.142;9.973) | 0.843 |
| **Distance to local facilities** |  |  |  |  |  |  |  |  |  |  |  |  |
| Cross-sectional^e^ | -10.743 | (-25.067;3.582) | 0.141 | **-12.224** | **(-22.512;-1.935)** | **0.020** | -21.312 | (-63.904;21.280) | 0.318 | **-11.762** | **(-21.642;-1.882)** | **0.020** |
| Longitudinal^f^ | 0.438 | (-27.286;-28.161) | 0.975 | 5.831 | (-15.045;26.707) | 0.583 | -8.760 | (-83.033;65.513) | 0.813 | -3.054 | (-22.919;16.811) | 0.763 |
| **Cycling infrastructures** |  |  |  |  |  |  |  |  |  |  |  |  |
| Cross-sectional^e^ | 0.129 | (-11.241;11.498) | 0.982 | 5.863 | (-3.482;15.208) | 0.218 | 14.924 | (-24.944;54.792) | 0.456 | 4.725 | (-4.421;13.871) | 0.310 |
| Longitudinal^f^ | 10.846 | (-11.923;33.615) | 0.349 | 11.005 | (-7.751;29.761) | 0.249 | -1.958 | (-69.102;65.186) | 0.954 | 5.886 | (-11.991;23.762) | 0.518 |
| **Walking infrastructures** |  |  |  |  |  |  |  |  |  |  |  |  |
| Cross-sectional^e^ | -3.809 | (-15.056;7.439) | 0.506 | 1.998 | (-6.864;10.860) | 0.658 | 8.786 | (-22.732;40.303) | 0.579 | 4.250 | (-4.290;12.789) | 0.329 |
| Longitudinal^f^ | 11.968 | (-10.928;34.864) | 0.304 | 12.171 | (-6.667;31.009) | 0.205 | 4.295 | (-58.638;67.229) | 0.892 | 5.245 | (-12.747;23.238) | 0.567 |
| **Total infrastructures** |  |  |  |  |  |  |  |  |  |  |  |  |
| Cross-sectional^e^ | -2.122 | (-14.021;9.777) | 0.726 | 4.807 | (-4.597;14.211) | 0.316 | 17.132 | (-21.523;55.788) | 0.378 | 5.549 | (-3.645;14.744) | 0.236 |
| Longitudinal^f^ | 11.353 | (-11.522;34.227) | 0.329 | 11.036 | (-7.843;29.914) | 0.251 | -2.016 | (-66.727;62.695) | 0.950 | 5.074 | (-12.890;23.039) | 0.579 |
| **Safety from crime** |  |  |  |  |  |  |  |  |  |  |  |  |
| Cross-sectional^e^ | -1.635 | (-13.461;10.191) | 0.786 | 0.129 | (-9.012;9.270) | 0.978 | -28.051 | (-67.875;11.774) | 0.164 | 0.402 | (-8.273;9.077) | 0.927 |
| Longitudinal^f^ | 9.926 | (-12.971;32.822) | 0.394 | 12.607 | (-6.151;31.364) | 0.187 | 5.691 | (-53.874;65.255) | 0.849 | 6.730 | (-11.219;24.679) | 0.462 |
| **Safety from traffic** |  |  |  |  |  |  |  |  |  |  |  |  |
| Cross-sectional^e^ | 2.723 | (-8.094;13.539) | 0.621 | 7.706 | (-0.324;15.735) | 0.060 | -6.395 | (-38.973;26.182) | 0.695 | 6.889 | (-0.984;14.762) | 0.086 |
| Longitudinal^f^ | 11.368 | (-11.427;34.162) | 0.327 | 13.736 | (-4.801;32.273) | 0.146 | 0.504 | (-65.026;66.034) | 0.988 | 7.616 | (-10.181;25.414) | 0.401 |
| **Total safety** |  |  |  |  |  |  |  |  |  |  |  |  |
| Cross-sectional^e^ | 0.917 | (-10.242;12.076) | 0.872 | 5.631 | (-3.068;14.330) | 0.204 | -17.902 | (-55.026;19.223) | 0.338 | 5.010 | (-3.370;13.389) | 0.241 |
| Longitudinal^f^ | 10.633 | (-12.230;33.495) | 0.361 | 13.859 | (-4.812;32.530) | 0.145 | -0.628 | (-63.146;61.889) | 0.984 | 7.672 | (-10.222;25.565) | 0.400 |
| **Esthetics** |  |  |  |  |  |  |  |  |  |  |  |  |
| Cross-sectional^e^ | 1.013 | (-11.261;13.287) | 0.871 | 3.391 | (-5.736;12.519) | 0.466 | -8.872 | (-48.884;31.139) | 0.658 | 6.182 | (-2.381;14.745) | 0.157 |
| Longitudinal^f^ | 10.931 | (-11.758;33.620) | 0.344 | 14.506 | (-4.105;33.117) | 0.126 | 4.454 | (-61.667;70.575) | 0.893 | 8.571 | (-9.278;26.421) | 0.346 |
| **Pleasure** |  |  |  |  |  |  |  |  |  |  |  |  |
| Cross-sectional^e^ | 2.274 | (-10.212;14.761) | 0.720 | 5.997 | (-3.082;15.076) | 0.195 | -13.894 | (-51.109;23.320) | 0.457 | 7.922 | (-0.651;16.495) | 0.070 |
| Longitudinal^f^ | 11.117 | (-11.586;33.819) | 0.336 | 14.614 | (-3.949;33.176) | 0.122 | 7.274 | (-57.104;71.652) | 0.822 | 8.753 | (-9.066;26.571) | 0.335 |
| **Connectivity** |  |  |  |  |  |  |  |  |  |  |  |  |
| Cross-sectional^e^ | 1.593 | (-9.449;12.635) | 0.777 | 4.017 | (-4.356;12.391) | 0.346 | 10.390 | (-28.109;48.890) | 0.591 | 4.982 | (-3.329;13.294) | 0.239 |
| Longitudinal^f^ | 10.528 | (-13.097;34.152) | 0.381 | 11.589 | (-7.218;30.397) | 0.226 | -12.328 | (-98.458;73.802) | 0.775 | 4.961 | (-13.188;23.109) | 0.591 |
| **Walking and cycling network** |  |  |  |  |  |  |  |  |  |  |  |  |
| Cross-sectional^e^ | -1.855 | (-13.163;9.452) | 0.747 | 3.217 | (-5.570;12.005) | 0.472 | 9.727 | (-25.571;45.025) | 0.583 | 2.209 | (-6.319;10.736) | 0.611 |
| Longitudinal^f^ | 12.333 | (-11.135;35.800) | 0.302 | 12.049 | (-6.775;30.872) | 0.209 | -9.630 | (-90.041;70.780) | 0.811 | 6.212 | (-11.950;24.375) | 0.502 |
| Values in bold are statistically significant (P <0.05) ; The treatment sub-groups were not mutually exclusive ; ^a^ Environmental scores were calculated from the ALPHA questionnaire (for Assessing Levels of PHysical Activity and Fitness at population level) ; ^b^ Self-reported physical activity was calculated from the Recent Physical Activity Questionnaire (RPAQ). The average difference in the outcome self-reported physical activity is expressed by the square root ; ^c^ 6MWD was measured by the 6-Minute Walk Test (6MWT). The average difference in the outcome 6MWD is expressed without transformation ; ^d^ The β indicate the overall longitudinal difference in the outcome score using linear mixed models per 1 SD of perceived built environment score after a standardized Z-score transformation. Analyses were adjusted on: age, social deprivation, educational level, employment status after diagnosis, comorbidities, living with a partner, trial arm, municipality class (except for Residential density score analyses), perceived home environment, COVID-19 pandemic trial status, longitudinal BMI, longitudinal quality of life, and longitudinal health status ; ^e^ The cross-sectional association of perceived neighborhood environment and physical activity is estimated by the environmental perception score term ; ^f^ The longitudinal association of perceived neighborhood environment and physical activity is estimated by the interaction term between the intervention visit and the environmental perception score ; ^d^The longitudinal association of perceived neighborhood environment and physical activity is estimated by the interaction term between the intervention visit and the environmental perception score. | | | | | | | | | | | | |
